# Mapping quantitative trait loci associated with leaf rust resistance in five spring wheat populations using single nucleotide polymorphism markers

**DOI:** 10.1371/journal.pone.0230855

**Published:** 2020-04-08

**Authors:** Firdissa E Bokore, Ron E. Knox, Richard D. Cuthbert, Curtis J. Pozniak, Brent D. McCallum, Amidou N’Diaye, Ron M. DePauw, Heather L. Campbell, Catherine Munro, Arti Singh, Colin W. Hiebert, Curt A. McCartney, Andrew G. Sharpe, Asheesh K. Singh, Dean Spaner, D. B. Fowler, Yuefeng Ruan, Samia Berraies, Brad Meyer

**Affiliations:** 1 Swift Current Research and Development Center, Agriculture and Agri-Food Canada, Swift Current, Canada; 2 Department of Plant Sciences, University of Saskatchewan, Saskatoon, Canada; 3 Morden Research and Development Centre, Agriculture and Agri-Food Canada, Morden, Canada; 4 Advancing Wheat Technologies, Swift Current, Canada; 5 Plant and Food Research, Canterbury Agriculture and Science Centre, Lincoln, New Zealand; 6 Department of Agronomy, Iowa State University, Ames, IA, United States of America; 7 Global Institute for Food Security, University of Saskatchewan, Saskatoon, Canada; 8 Department of Agricultural, Food and Nutritional Science, 4–10N Agriculture-Forestry Centre, University of Alberta, Edmonton, Canada; Murdoch University, AUSTRALIA

## Abstract

Growing resistant wheat (*Triticum aestivum* L) varieties is an important strategy for the control of leaf rust, caused by *Puccinia triticina* Eriks. This study sought to identify the chromosomal location and effects of leaf rust resistance loci in five Canadian spring wheat cultivars. The parents and doubled haploid lines of crosses Carberry/AC Cadillac, Carberry/Vesper, Vesper/Lillian, Vesper/Stettler and Stettler/Red Fife were assessed for leaf rust severity and infection response in field nurseries in Canada near Swift Current, SK from 2013 to 2015, Morden, MB from 2015 to 2017 and Brandon, MB in 2016, and in New Zealand near Lincoln in 2014. The populations were genotyped with the 90K Infinium iSelect assay and quantitative trait loci (QTL) analysis was performed. A high density consensus map generated based on 14 doubled haploid populations and integrating SNP and SSR markers was used to compare QTL identified in different populations. AC Cadillac contributed QTL on chromosomes 2A, 3B and 7B (2 loci), Carberry on 1A, 2B (2 loci), 2D, 4B (2 loci), 5A, 6A, 7A and 7D, Lillian on 4A and 7D, Stettler on 2D and 6B, Vesper on 1B, 1D, 2A, 6B and 7B (2 loci), and Red Fife on 7A and 7B. Lillian contributed to a novel locus *QLr*.*spa-4A*, and similarly Carberry at *QLr*.*spa-5A*. The discovery of novel leaf rust resistance QTL *QLr*.*spa-4A* and *QLr*.*spa-5A*, and several others in contemporary Canada Western Red Spring wheat varieties is a tremendous addition to our present knowledge of resistance gene deployment in breeding. Carberry demonstrated substantial stacking of genes which could be supplemented with the genes identified in other cultivars with the expectation of increasing efficacy of resistance to leaf rust and longevity with little risk of linkage drag.

## Introduction

Leaf rust, caused by *Puccinia triticina* Eriks., is an economically devastating fungal pathogen threatening wheat (*Triticum aestivum* L.) production worldwide [1–4]. Leaf rust occurs more regularly and in more regions world-wide than stem rust (*P*. *graminis*) or stripe rust (*P*. *striiformis*) of wheat [5]. Many studies indicate that *P*. *triticina* spores travel long distances by wind or man, and cause damage to wheat beyond their country of origin. For example, in North America several studies [2, 5, 6] indicated the disease establishes in the fall on winter wheats that are grown in the southern USA, and travels by winds the following spring and summer to the northern USA and Canada along the “*Puccinia* pathway”.

Growing resistant wheat varieties is an important method for control of leaf rust by farmers because input costs are minimized with reduced requirement of fungicides while environmental sustainability is improved [3]. However, achieving durable resistance can be difficult as the rust pathogen continues to evolve and overcome major genes that have been deployed [2, 7]. In addition to evolution of new races, exotic incursions of rust pathogen races have occurred in recent decades and pose a threat to wheat production areas on different continents [2].

Two types of resistance, seedling (or all-stage) resistance and adult plant resistance (APR), to wheat rusts are known [8]. Most seedling resistance genes are effective from the early seedling stage throughout the life of the plant and are characterized by race specificity and low infection types; whereas adult plant resistance is largely effective at the adult plant growth stage [3, 8]. Seedling resistance is typically monogenic and has been favoured in breeding because of the high level of expressivity and simplicity of phenotypic selection, but most genes have been overcome by the emergence of virulent races. This is illustrated in the case of spring wheat in Canada. The most common leaf rust resistance genes in Canada Western Red Spring (CWRS) wheat varieties are *Lr1*, *Lr10*, *Lr13*, *Lr14a*, *Lr16*, *Lr21* and *Lr34* [3, 9]. Renown was the first wheat variety to be widely grown in Canada having the resistance gene *Lr14a*, but virulence to the gene was common by 1945 due to changes in the *P*. *triticina* population [3]. Subsequently *Lr16* was deployed but lost its effectiveness [3]. Next the *Lr21* gene, which has been cloned [10], succumbed to virulence in 2010 in the United States and in Canada in 2011 [11]. Furthermore genes *Lr1*, *Lr10* and *Lr13* are no longer effective [12]. Ultimately the majority of known all-stage resistance genes have been defeated [13–15] leaving few genes for deployment in resistance breeding.

Adult plant resistance genes such as *Lr34*, *Lr46* and *Lr67* [13–15] have also been used along with resistance from seedling genes. Other well characterized leaf rust adult plant resistance genes include *Lr68*, *Lr74*, *Lr75*, *Lr77*, and *Lr78* [16]. In some cases, these genes work synergistically with seedling resistance such as *Lr10*, *Lr13*, *Lr16*, and *Lr18* which confer resistance in combination with other genes, particularly *Lr34* [3, 17]. Combinations of adult plant resistance genes and other minor effect genes that condition resistance to a broad spectrum of *P*. *triticina* races are key to the development of wheat varieties with long-lasting resistance to leaf rust [18].

Mapping resistance genes in existing adapted parental stocks is necessary in order to understand the effective gene combinations and for efficient marker assisted selection [19]. Recent molecular mapping studies uncovered several quantitatively inherited sources of leaf rust resistance based on minor effect genes in different wheat germplasm. For example, in their review of research from the last fifteen years, Li et al. [20] documented 80 leaf rust resistance QTL involving sixteen wheat chromosomes. A few years later, in their review work, Da Silva, Zanella [21] reported 249 leaf rust resistance QTL identified in 70 bi-parental populations and 79 donor lines. Modern Canadian spring wheat cultivars Lillian, Stettler, Carberry, AC Cadillac, Vesper and a founder cultivar Red Fife show varying levels of leaf rust resistance, and although Singh et al. [22] studied leaf rust resistance in cultivars Carberry and AC Cadillac, the genes providing resistance have not been fully characterized. This study sought to identify the chromosomal locations and effects of genes controlling leaf rust resistance in spring wheat cultivars Lillian, Stettler, Carberry, AC Cadillac, Vesper and Red Fife.

## Materials and methods

### Plant materials

Five doubled haploid (DH) populations—Carberry/AC Cadillac, Carberry/Vesper, Vesper/Lillian, Vesper/Stettler and Stettler/Red Fife—were generated by the maize pollen method [23] from F_1_ plants of crosses between cultivars of the market class Canada Western Red Spring (CWRS), including a founder cultivar Red Fife. The pedigree descriptions and leaf rust resistance genes possibly possessed by these parents are described in S1 Table. The number of lines evaluated in each of the five populations ranged from 94 to 775 (S2 Table). The number of lines phenotyped per population varied for different reasons mainly based on the number of lines available from the doubled haploid system, cost of genotyping, or taking advantage of phenotyping a subset of the population used for a different study. The lines used for genotyping were chosen randomly and became the basis of the population size for QTL analysis.

### Rust infection phenotyping under field conditions

Testing of the populations was done as described by Bokore et al. [24] and Singh et al. [22]. Briefly, the populations were grown in un-replicated single row plots and assessed for leaf rust severity and infection response in disease nurseries in Canada near Swift Current, SK from 2013 to 2015, Morden, MB from 2015 to 2017, Brandon, MB in 2016 and in New Zealand near Lincoln in 2014 (S2 Table). Parents and check cultivars were repeated in each experiment and spreader rows of susceptible genotypes were planted around the plots to enhance disease development.

Inoculum of *P*. *triticina* was generated by increasing urediniospores of all races in the proportions that they were found in western Canada in the year prior to the field trial. The frequency of virulence to 16 leaf rust resistance genes in these population mixes is shown in Table 1. Urediniospores of these multi-race mixtures were used to inoculate spreader rows susceptible to leaf rust at the Swift Current, Morden and Brandon locations. This inoculum was generated by increasing and collecting urediniospores from a representative mixture of the virulence phenotypes found in Canada during the annual national virulence survey in the previous year [25]. For each year, all the isolates generated during the virulence survey of Manitoba and Saskatchewan were combined to generate this field inoculum. Each year, 18 to 64 unique virulence phenotypes were included in this field inoculum (Table 1). In a given season, the same *P*. *triticina* race composition was used in Morden, Swift Current and Brandon trials. At the Morden and Brandon locations urediniospores were suspended in light mineral oil (Soltrol, Chevron Phillips Chemical Co) and sprayed on the leaves of the spreader rows at early tillering, subsequently leaf rust developed on the spreader rows and urediniospores were windblown to the test lines to provide infection. At Swift Current, spreader rows of susceptible genotypes were needle inoculated with urediniospores. At Lincoln, natural infection was the sole source of inoculum. No artificial inoculation was carried out.

**Table 1:** Frequency of virulence to 16 leaf rust resistance genes in the *Puccinia triticina* inoculum mixture used to inoculate field screening nurseries in Canada between 2010 and 2016.

Year	Number of isolates	Number of virulence phenotypes	Gene																
			*Lr1*	*Lr2a*	*Lr2c*	*Lr3*	*Lr9*	*Lr16*	*Lr24*	*Lr26*	*Lr3ka*	*Lr11*	*Lr17*	*Lr30*	*LrB*	*Lr10*	*Lr14a*	*Lr18*	*Lr21*
2010	341	18	100	48.1	49.9	100	31.7	0	60.4	10	18.2	1.5	51.9	10.6	51.9	100	78.3	0	0
2011	216	33	100	52.3	63.6	100	12.1	2.8	60.7	13.1	20.1	0.9	47.7	15.4	46.7	99.5	57.9	0	7
2012	177	28	100.0	62.1	67.8	100.0	24.9	4.0	52.0	7.3	14.1	0.0	38.4	10.2	40.1	100.0	47.5	2.3	10.2
2013	236	29	100	42.8	43.2	100	33.5	0	41.1	10.6	28	5.9	56.8	26.3	56.8	96.2	66.9	0	10.6
2014	93	29	100	55.8	62.1	98.9	28.4	2.1	24.2	13.7	24.2	7.4	62.1	24.2	46.3	93.7	45.3	1.1	2.1
2015	208	42	99	41.3	41.8	99.5	53.8	4.3	48.6	24.5	37	5.3	62.5	36.5	63.9	98.1	73.1	0.5	10.1
2016	233	64	100	24.9	26.2	100	51.5	16.7	63.1	29.2	47.6	3.9	73.8	45.9	77.7	98.7	83.7	0	4.3

The percent leaf rust severity of infected flag leaves was scored using the modified Cobb Scale [26] at all locations except Lincoln in 2014 where a scale of 0 to 10 was used and converted to percent by multiplying values by 10. Infection response was recorded as resistant (R), resistant to moderately resistant (RMR), moderately resistant (MR), mesothetic (X), moderately resistant to moderately susceptible (MRMS), moderately susceptible (MS), moderately susceptible to susceptible (MSS), and susceptible (S). Infection response was not recorded for trials planted at Lincoln. The infection response scores were converted into numeric values based on R = 1, RMR = 2, MR = 3, X = 4, MRMS = 5, MS = 6, MSS = 7, and S = 8 for QTL analysis.

### Genotyping, construction of linkage maps and QTL analysis

The DNA of parents and DH lines was extracted from young leaves with the BioSprint 96 DNA Plant Kit (QIAGEN Science, Maryland, USA). Table 2 shows the number of DH lines genotyped with the 90K Infinium iSelect SNP wheat assay (Illumina Inc., San Diego, CA). In addition, SNP12, a co-dominant Lr34 diagnostic SNP marker modified from the dominant marker caSNP12 [27] on chromosome 7D was integrated into the maps of Carberry/Vesper and Vesper/Lillian populations. *SNP12* is defined with primer sequences AAG AAT GAA GCC TCC GAA TG (forward) and CAT TCA GTC ACC TCG CAG (reverse). The *SNP12* assay was performed on the Roche LightCycler II Real-Time Thermal Cycler using the High Resolution Melt (HRM) module (S3 Table). During PCR, a 117 base amplicon is amplified containing the SNP nucleotide. Because the amplicon is monomorphic in size for all samples, polymorphism cannot be detected via electrophoresis. Therefore, the additional step of HRM analysis is performed to detect the differences in melt curve signatures caused by the differing SNP base after PCR is complete.

**Table 2:** Number of lines, number of linkage groups, number of markers, genomic size and map density of five doubled haploid mapping populations used in the leaf rust resistance QTL analysis.

Population name	Number of doubled haploid lines	Number of linkage groups	Number of markers	Length (cM)	Density
Carberry/AC Cadillac	775	29	6806	3237.9	0.7
Carberry/Vesper	188	28	6138	1835.4	0.3
Vesper/Lillian	283	29	7839	3679.5	0.8
Vesper/Stettler	94	22	4989	2002.0	0.4
Stettler/Red Fife	218	26	9983	3247.6	0.3

Genetic maps were built for each of the five populations, using the two-step mapping strategy described previously [28, 29]. Briefly, ‘draft’ linkage maps for individual populations were generated using the minimum spanning tree map (MSTMap) software using a stringent cut off p-value of 1E^-10^ and a maximum distance between markers of 15 cM. Then, the ‘draft’ maps were refined using the MapDisto version 1.7.5 software using a cut off recombination value of 35%, a minimum LOD score of 3.0 and the Kosambi mapping function. The best order of markers was generated using both “AutoCheckInversions” and “AutoRipple” commands. Then, we built a consensus map based on fourteen hexaploid wheat mapping populations including the five QTL populations (Carberry/AC Cadillac, Carberry/Vesper, Vesper/Lillian, Stettler/Vesper and Stettler/Red fife) used in the current leaf rust resistance mapping study and nine others, namely: 8021V2/AC Karma [30], AAC Concord/CDC Hughes, Attila/CDC Go [31], Carberry/Thatcher, Cutler/AC Barrie [28], Norstar/Capelle Despres [29], Norstar/Manitou [29], Norstar/Winter Manitou [29] and RL4452/AC Domain. In addition to SNP markers, 8021V2/AC Karma was genotyped with 529 microsatellite or simple sequence repeat (SSR) [32–34] markers. The individual genetic maps were integrated into a consensus map, using the LPmerge R package [35, 36]. This software uses a linear programming algorithm to minimize the mean absolute error between the consensus map and the individual maps. For the goodness-of-fit for the consensus map, LPmerge computes a root-mean-square error (RMSE) per linkage group by comparing the position (in cM) of all markers on the consensus map with that on the individual maps. This metric was calculated for different maximum interval sizes (k in the algorithm), ranging from 1 to 10. The value of k minimizing the mean RMSE per linkage group was selected for construction of the consensus map. The consensus map integrated both SNP and SSR markers.

Quantitative trait loci analysis (QTL) was applied to all population x environment combinations for leaf rust severity and infection response except for Carberry/Vesper at Lincoln in 2014 in which disease development was insufficient to discriminate among the lines. QTL analyses were performed on five rust evaluated bi-parental crosses whereas the consensus map based on fourteen populations was used only for comparing QTL positions. The leaf rust resistance loci were identified by performing QTL analysis using MapQTL.6^®^ [37]. The permutation test option (1000 permutations) within MapQTL was applied to determine the significant threshold for the logarithm of the odds (LOD). Genome-wide threshold levels were used to declare significant QTL at the 5% level of significance. Automatic co-factor detection based on backward elimination to identify the co-factor markers as well as manual co-factor selection was performed for Multiple QTL Mapping (MQM). The position in the genetic map of the SNP markers associated with each of the QTL were aligned with the hexaploid consensus map using MapChart [38] to investigate (1) the relationship of the QTL identified in different cultivars, and (2) if the QTL are located in the same region with those reported by other studies.

A microsatellite marker *wmc44* associated with the leaf rust gene *Lr46* [39, 40] and co-located to chromosome 1BL, was run on Carberry/Vesper and Vesper/Lillian populations to see the similarity of a QTL identified on chromosome 1BL and derived from Vesper in the Vesper/Lillian population. As *Lr46* was originally reported in Pavon 76 and Lalbahadur (a monosomic line carrying *Lr46* [15]), the two lines were genotyped by a Kompetitive Allele Specific PCR (KASP) marker converted from SNP marker probes associated with the 1BL QTL in Vesper in the Vesper/Lillian population to investigate if the 1BL QTL corresponds with the *Lr46* gene.

## Results

### Leaf rust reaction

**Fig 1:**
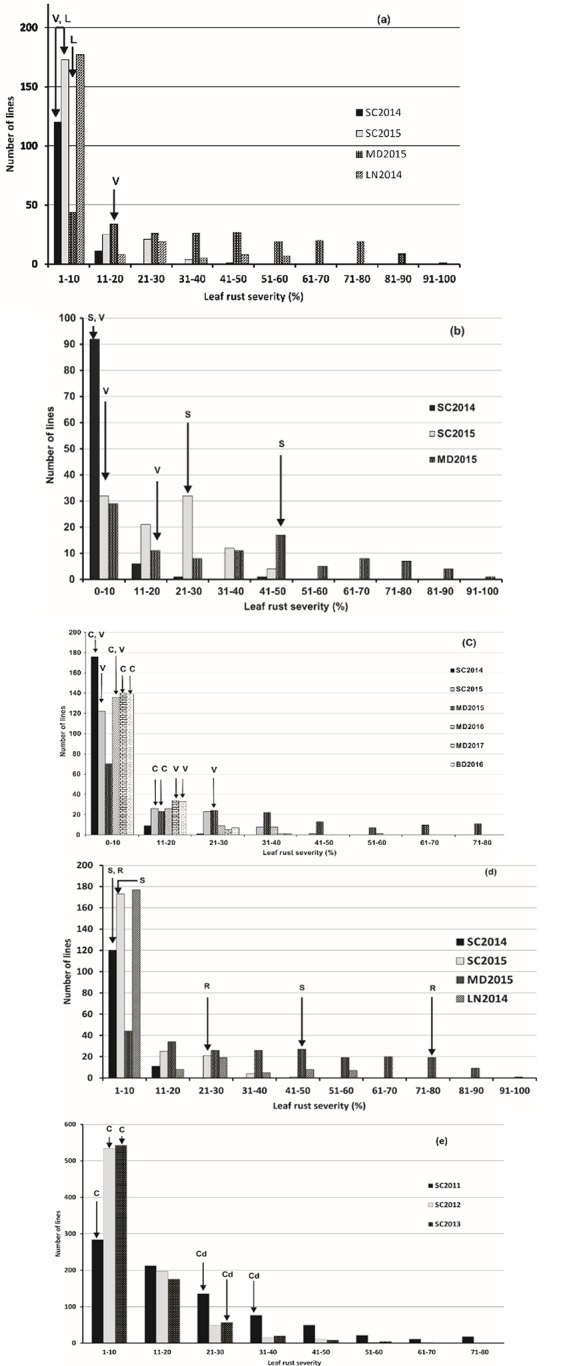
Frequency distribution of five doubled populations (a) Vesper/Lillian, (b) Vesper/Stettler, (c) Carberry/Vesper, (d) Stettler/Red Fife, and (e) Carberry/AC Cadillac for adult plant leaf rust severity. Arrows indicate parent leaf rust severity: C, Carberry; Cd, AC Cadillac; V, Vesper; L, Lillian; S, Stettler; Rf, Red Fife. In the key, the test year is preceded by the location defined as follows: SC, Swift Current, MD, Morden, Canada, and LN, Lincoln, New Zealand.

The response to leaf rust of parents of the populations varied in different environments (Fig 1, S4 Table). The greatest amount of leaf rust occurred at Morden in 2015. In this environment, Lillian was the most resistant cultivar among the parents and displayed 3% disease severity, while Red Fife with a severity of 78% was the most susceptible. The highest reaction scored on Carberry at 15% severity, Vesper at 30% and Stettler at 60% was observed in the Morden 2015 environment. The Carberry/AC Cadillac population was not planted at Morden, but Carberry displayed lower disease severity than AC Cadillac in the Swift Current environments with adequate levels of disease. The infection responses of the cultivars paralleled the severities, with Lillian, Vesper, Carberry and AC Cadillac varying between R and MRMS, and that of Stettler and Red Fife varying from MR to S.

The distributions of leaf rust severity were continuous for all the populations, and lines transgressive for resistance appeared occasionally in the resistant tail and regularly in the susceptible tail (Fig 1). The populations generally displayed skewed distributions with a preponderance of lines showing resistance to leaf rust. Considering all the test environments, the greatest range in severity of the lines was with the Vesper/Lillian (1–95%), Vesper/Stettler (1–95%) and Stettler/Red Fife (1–95%) populations, while the Carberry/Vesper was somewhat narrower (0–80%) as was Carberry/AC Cadillac (1–80%).

### Genetic maps

Individual high-density maps were built for the five populations phenotyped for leaf rust in this QTL mapping study. The number of markers, genomic size as map length in cM, and map density of the populations used for consensus map development are shown in S5 Table and for leaf rust QTL analysis in Table 2 along with number of linkage groups generated for the latter. For example, for the Vesper/Lillian population, the map consisted of 29 linkage groups and 7839 markers that spanned 3679.5 cM. The consensus map we built comprising of SNP and SSR markers using 14 mapping populations allowed comparison of our research results with previous reports on leaf rust resistance genes.

S1 File presents the hexaploid wheat consensus map and genetic maps of the 14 different populations used to build the consensus map. Table 3 demonstrates some of the features of the hexaploid wheat consensus map. It consisted of 36715 SNP and SSR markers spanning all hexaploid wheat chromosomes and covered a length of 3162 cM with an average marker spacing of 0.13 cM/marker. The D genome covered the smallest map length at 642 cM, with the lowest average marker density of 6.4 markers/cM, while A genome marker density was 11.3 markers/cM and the B genome 14.6 markers/cM.

**Table 3:** Statistics characterizing totals as well as individual chromosomes of the consensus map built from 14 hexaploid wheat populations^a^: Chromosome name, number of markers, map length (cM), map density and average marker spacing.

Chromosome	Number of markers	Length (cM)	Map density (cM/Marker)	Marker density (Marker/cM)
1A	2526	186.37	0.07	13.6
2A	1978	187.69	0.09	10.5
3A	1673	194.13	0.12	8.6
4A	1630	169.63	0.1	9.6
5A	1795	218.33	0.12	8.2
6A	2685	151.0	0.06	17.8
7A	2502	198.55	0.08	12.6
**Total A Genome**	**14789**	**1305.7**	**0.09**	**11.3**
1B	2450	166.95	0.07	14.7
2B	3849	199.06	0.05	19.3
3B	2371	154.36	0.07	15.4
4B	1294	153.77	0.12	8.4
5B	3018	197.45	0.07	15.3
6B	2702	206.8	0.08	13.1
7B	2108	137.29	0.07	15.4
**Total B Genome**	**17792**	**1215.68**	**0.08**	**14.6**
1D	762	85.85	0.11	8.9
2D	1291	119.22	0.09	10.8
3D	886	122.46	0.14	7.2
4D	120	71.98	0.6	1.7
5D	412	69.11	0.17	6.0
6D	398	83.39	0.21	4.8
7D	265	89.51	0.34	3.0
**Total D Genome**	**4134**	**641.52**	**0.24**	**6.4**
Total	36715	3162.9	0.13	11.6

^a^ The 14 populations are derived from crosses between 8021V2/AC Karma, AAC Concord/CDC Hughes, Attila/CDC Go, Carberry/Thatcher, Cutler/AC Barrie, Norstar/Capelle Despres, Norstar/Manitou, Norstar/Winter Manitou and RL4452/AC Domain

### QTL analysis

Twenty leaf rust resistance QTL were identified in the five populations. In S1 Fig, the positions of the QTL in each population were aligned with the hexaploid wheat consensus map we generated. A summary of markers with the highest LOD scores at each QTL, phenotypic variation explained, and associated additive effects is presented in Table 4. The source of resistance and environments that revealed the QTL are listed in S6 Table, whereas the detailed information on each QTL is given in S7 Table.

**Table 4:** Closest marker, associated LOD score, percentage of phenotypic variation explained by individual QTL and the level of additive effect of QTL identified in five doubled haploid populations evaluated for their field responses against leaf rust in nurseries near Swift Current, SK, Morden and Brandon, MB, Canada and Lincoln, New Zealand.

QTL	Closest marker	Position, cM	Trait^a^	LOD^b^	PVE(%)^c^	Add^d^	LOD	PVE(%)	Add	LOD	PVE(%)	Add	LOD	PVE(%)	Add	LOD	PVE(%)	Add	LOD	PVE(%)	Add	Source^e^
***Carberry/AC Cadillac*** (4^f^)				Swift Current 2011			Swift Current 2012			Swift Current 2013			Swift Current 2014									
*QLr*.*spa-1A*	*IACX1465*	42.7	Sev	8.4	4.8	9.6	15.1	8.6	7.3	11.6	6.7	7.1										C
			IR	3.4	2.0	0.6	3.8	2.2	0.5	4.0	2.3	0.4										C
*QLr*.*spa-2A*.*1*	*BS00041816_51*	50.0	Sev	4.6	2.7	-2.9	4.2	2.4	-1.6	7.1	4.1	-2.3										Cd
			IR	10.2	1.8	-0.3	10.1	5.8	-0.2	7.2	4.2	-0.3										Cd
*QLr*.*spa-2B*.*1*	*Excalibur_c39493_251*	7.4	Sev	3.4	2.0	4.7																C
			IR				3.3	1.9	0.3													C
*QLr*.*spa-2B*.*2*	*Kukri_c53810_137*	38.8	Sev	6.4	3.6	8.3	10.6	5.9	6.1	15.5	8.7	8.2										C
			IR	3.5	2.0	0.6	3.8	2.2	0.2	3.3	1.8	0.4										C
*QLr*.*spa-2D*.*1*	*Ex_c2115_3369*	81.7	Sev	3.9	2.3	2.7	3.4	2.0	1.5													C
			IR																			
*QLr*.*spa-3B*	*Tdurum_contig79629_538*	15.1	Sev	2.1	1.2	-2.0				4.0	2.3	-1.7										Cd
			IR	2.6	1.5	-0.2				4.8	2.8	-0.2										Cd
*QLr*.*spa-4B*.*1*	*Tdurum_contig12204_1131*	0.0	Sev	3.4	1.9	2.5	3.3	1.9	1.4	3.5	2.0	1.6										C
			IR	3.0	1.7	0.2	5.3	3.0	0.3	4.5	2.5	0.2										C
*QLr*.*spa-4B*.*2*	*BS00021984_51*	75.7	Sev	4.5	2.6	2.9	3.3	1.9	1.4	3.0	1.7	1.5										C
			IR	5.2	3.0	0.3	6.3	3.6	0.3	6.7	3.8	0.3										C
*QLr*.*spa-5A*	*BobWhite_c1387_798*	41.6	Sev	4.5	2.6	3.1	7.9	4.6	2.3	9.4	5.4	2.8	3.8	7.3	1.8							C
			IR	4.8	2.8	0.2	3.1	1.8	0.1	6.5	3.8	0.3										C
*QLr*.*spa-6A*	*BobWhite_c39821_195*	4.2	Sev				11.5	6.6	6.4	14.7	8.3	8.0										C
*QLr*.*spa-7A*	*BS00063860_51*	193.3	Sev							3.8	2.2	1.7										C
*QLr*.*spa-7B*.*1*	*Ex_c101666_634*	31.5	Sev	9.2	5.2	-4.1	15.5	8.6	-3.0	18	9.9	-3.6	2.8	4.5	-1.4							Cd
			IR	12.4	7.0	-0.5	14.9	8.5	-0.3	18.2	10.2	-0.4	5.8	10.7	-0.4							Cd
*QLr*.*spa-7B*.*2*	*RAC875_c57326_85*	145.9	Sev	4.5	2.4	-7.4	21.3	10.6	-8.8	15.2	7.6	-8.3										Cd
			IR							3.0	1.6	-0.4										Cd
***Carberry/Vesper*** (7^g^)				Swift Current 2014			Swift Current 2015			Morden 2015			Morden 2016			Morden 2017			Brandon 2016			
*QLr*.*spa-1D*	*RAC875_c2070_566*	67.5	Sev	6	13.8	1.6	6.7	16	4.5	9.7	21.9	11							2.9	7.1	1.9	V
			IR	5	11.6	0.4	5.5	12	1.3	8.4	19.4	1.2										V
*QLr*.*spa-2A*.*2*	*Kukri_c46040_620*	0.0	Sev																3.6	8.8	2.1	V
			IR							4.7	11.3	0.9										V
*QLr*.*spa-2B*.*1*	*BobWhite_c12144_216*	0.0	Sev							2.5	6.1	-5.8	2.8	6.8	-2.8	3.6	8.8	-2.2	2.5	6.3	-1.7	C
			IR										2.7	6.7	-0.4	3.3	8.2	-0.5				C
*QLr*.*spa-7A*	*BS00053365_51*	99.8	Sev	3.0	2.4	-1.1	7.6	3.0	-3.1													C
			IR				2.6	2.6	-0.7													C
*QLr*.*spa-7D*	*SNP12*	2.0	Sev							7.5	17.5	-9.8	5.9	14	-4	9.9	22.4	-3.5	8	18.5	-3	C
			IR	3.3	7.9	-0.3	3.6	8.7	-0.8	6.5	15.4	-1.1	5.4	12.9	-0.6	7.7	18	-0.7	7	16.5	-0.6	C
***Vesper/Lillian*** (5)				Swift Current 2013			Swift Current 2014			Swift Current 2015			Morden 2015			Lincoln 2014						
*QLr*.*spa-1B*	*wsnp_Ex_c1058_2020681*	181.9	Sev				2.2	3.7	2.2													V
			IR				3	4.9	0.5													
*QLr*.*spa-1D*	*Kukri_c2408_784*	1.0	Sev	23.5	34.1	8.4	13.2	20	5.1	4.4	6.9	3.4	22.7	31.1	17.5	21.9	30.4	1.3				V
			IR	16	24.7	1.2	15.8	24	1.2	15.5	22.6	1.7	19.6	27.5	1.7							V
*QLr*.*spa-4A*	*Ex_c70424_465*	73.3	Sev	3.8	4.1	-3.1	3	5	-2.6	4.7	7	-4.7	3.3	5.2	-20	6.1	9	-0.8				L
			IR	4.3	6.6	-1.7	4.4	6.6	-0.7	3.6	5.8	-0.9	3.6	5.6	-2.1							L
*QLr*.*spa-6B*	*BobWhite_c36415_378*	118.6	Sev	3.8	6.6	3.7	4.7	7.7	3.2							3.9	6.2	0.6				V
*QLr*.*spa-7B*.*1*	*Kukri_c109962_396*	0.0	Sev	4.2	3.7	6.7																V
			IR	3	5.1	0.6																V
*QLr*.*spa-7B*.*2*	*RFL_Contig71_386*	232	Sev	4.9	7.7	4.1	5.1	3.3	8.1	4.8	7.3	3.5	6.5	9.8	9.8							V
			IR										5	7.6	0.9							V
*QLr*.*spa-7D*	*SNP12*	7.5	Sev	5.9	9.9	-4.6	3.4	5.7	-2.7	3.1	5	-0.8	8.4	12.9	-11							L
			IR	10	16.3	-1							6.4	10	-1							L
***Vesper/Stettler*** (5)							Swift Current 2014			Swift Current 2015			Morden 2015			Lincoln 2014						
*QLr*.*spa-1D*	*BobWhite_c4303_524*	58.6	S				5.1	22.4	3.1	7.2	29.6	8.5	12.5	44.8	18.9	3.3	15.1	5.4				V
			IR				4.2	19.2	0.9	4.6	23.5	1.1	6.7	28	1.5							V
*QLr*.*spa-2A*.*2*	*BS00022393_51*	50.1	Sev										3.5	15.7	11.2							V
			IR				3.4	13.6	0.7				3.2	14.4	1.1							V
***Stettler/Red Fife*** (4)							Swift Current 2014			Swift Current 2015			Morden 2015			Lincoln 2014						
*QLr*.*spa-2D*.*2*	*Kukri_rep_c105822_804*	59.1	Sev				3.3	10.5	1.1	12.9	24	4.8	21.1	36.1	16	6.8	13.3	0.5				S
			IR							14.3	26.2	1	22.5	37.9	1.7							S
*QLr*.*spa-6B*	*BS00010993_51*	111.1	Sev							3.9	8	2.8										S
*QLr*.*spa-7A*	*tplb0031i24_1212*	4.4	Sev										5.6	11.2	-8.9							R
			IR										4.1	8.4	-0.8							R
*QLr*.*spa-7B*.*2*	*BS00108630_51*	166	Sev													3.1	6.2	-0.3				R

^a^ Sev = leaf rust severity, IR = leaf rust infection response

^b^ Logarithm of odds (LOD) score

^c^ Percentages of phenotypic variation explained by individual QTL

^d^ Additive effect

^e^ Source of resistance allele: Cd, AC Cadillac; C, Carberry; L, Lillian; V, Vesper; S, Stettler; R, Red Fife

^f^ Total number of test environments per population

^g^ Tested in New Zealand in 2014 but no trait differential

Location in the International Wheat Genome Consortium (IWGC) Chinese spring wheat RefSeq. genome v.1.0 of the SNP markers that detected the leaf rust resistance QTL in the present study is presented in S8 Table. The QTL revealed from the results of mapping will be further elaborated upon on a population by population basis.

### Carberry/AC Cadillac population

Genetic mapping of the Carberry/AC Cadillac population resulted in the identification of 13 QTL—nine of them contributed by Carberry on chromosomes 1A (*QLr*.*spa-1A*), 2B (2 loci) (*QLr*.*spa-2B*.*1* and *QLr*.*spa-2B*.*2*), 2D (*QLr*.*spa-2D*.*1*), 4B (2 loci) (*QLr*.*spa-4B*.*1* and *QLr*.*spa-4b*.*2*), 5A (*QLr*.*spa-5A*), 6A (*QLr*.*spa-6A*) and 7A (*QLr*.*spa-7A*) and four by AC Cadillac on 2A (*QLr*.*spa-2A*.*1*), 3B (*QLr*.*spa-3B*) and 7B (2 loci) (*QLr*.*spa-7B*.*1* and *QLr*.*spa-7B*.*2*) (S1 Fig; Table 4 S6 and S7 Tables). The QTL *QLr*.*spa-1A* and *QLr*.*spa-2A*.*1* were detected in three of four environments in Canada. *QLr*.*spa-1A* was among the most effective loci explaining phenotypic variation approaching 9% in leaf rust severity although the infection response explained was lower at 2.3%. On the consensus map, several SSR markers (*barc28*, *gwm164*, *wmc278*, *wmc469*, and *gwm357*) are located nearby the SNP markers (*IACX1465* and *Excalibur_c46833_204*) that define *QLr*.*spa-1A*. *QLr*.*spa-2A*.*1* had more impact on infection response explaining up to 6% of the phenotypic variation than leaf rust severity at 4%. SNP markers anchoring *QLr*.*spa-2A*.*1* mapped in the region of *barc5*, *gwm294* and *wmc1709*.

Two apparent QTL were detected on chromosome 2B from Carberry. The first, *QLr*.*spa-2B*.*1* mapped adjacent to SSR markers *wwmc661*, *wmc382b*, *wmc764*, *wmc489a* and *barc35* (S1 Fig) and was detected in only two of four environments. This *QLr*.*spa-2B*.*1* allele from Carberry was additionally detected in the Carberry/Vesper population. The second 2B QTL, *QLr*.*spa-2B*.*2*, was detected in three out of four Canadian environments. *QLr*.*spa-2B*.*2* could be quite effective, accounting for up to 9% of the total variation in leaf rust severity although only 2% in infection response. The closet SNP marker to *QLr*.*spa-2B*.*1*, *Kukri_c53810_137*, was located 0.2–0.9 cM from SSR loci *wmc257*, *wmc25a* and *wmc154*.

The 2D QTL, *QLr*.*spa-2D*.*1*, was significantly associated with leaf rust severity in two environments, but not with infection response. Located on the long arm of the chromosome, this QTL is different from a major QTL, *QLr*.*spa-2D*.*2*, that was identified in Stettler on 2DS about 33 cM from *QLr*.*spa-2D*.*1*. Like *QLr*.*spa-2D*.*1*, the 3B QTL *QLr*.*spa-3B* was expressed in two environments albeit marginally in the one and was flanked by *UMN10* [41], *gwm389*, *bar147* and *xsts3B-142*.

*QLr*.*spa-4B*.*1* and *QLr*.*spa-4B*.*2* were about 75 cM from each other on chromosome 4B. Both QTL expressed in the same three of four Canadian environments. The explained phenotypic variation for these two QTL was relatively low ranging from 1.7% to 3.8%. Even more consistent than the 4B loci, *QLr*.*spa-5A* was detected in all four test environments with a moderately high phenotypic variation of up to 7.3% explained for leaf rust severity. In contrast, *QLr*.*spa-6A* was significant in two of the four tests and *QLr*.*spa-7A* in a single environment only. Although the effect of *QLr*.*spa-7A* was quite low, explaining 2.2% of leaf rust severity, *QLr*.*spa-6A* was more effective, explaining up to 8% of the severity.

*QLr*.*spa-7B*.*1* and *QLr*.*spa-7B*.*2* corresponded with two QTL detected in the Vesper/Lillian population. *QLr*.*spa-7B*.*2* was similarly detected in Red Fife in the Stettler/Red Fife population. Although both QTL displayed consistent and quite strong effects in the Carberry/AC Cadillac population, the response was more variable in the Vesper and Red Fife genetic backgrounds. The SNP markers that tagged *QLr*.*spa-7B*.*1* mapped with SSR marker loci *wmc323*, *wmc606*, *gwm537* and *wmc76*, while *QLr*.*spa-7B*.2 associated SNP markers mapped with *wmc581* and *gwm344b* (S1 Fig).

### Carberry/Vesper population

Five leaf rust resistance QTL were detected in the Carberry/Vesper population, from Carberry on chromosomes 2B (*QLr*.*spa-2B*.*2*), 7A (*QLr*.*spa-7A*) and 7D (*QLr*.*spa-7D*) and Vesper on 1D (*QLr*.*spa-1D*) and 2A (*QLr*.*spa-2A*.*2*) (S1 Fig; Table 4 S6 and S7 Tables). *QLr*.*spa-1D* was located in the same genomic region as the Vesper QTL detected in the other two Vesper populations Vesper/Lillian and Vesper/Stettler. The *QLr*.*spa-1D* associated SNP markers were located close to three SSR markers (*wmc432*, *barc149* and *gdm33b*) on the consensus map. The QTL was detected in four out of six Canadian environments and had a strong effect explaining up to 22% of the total variation in leaf rust severity and 20% in infection response. The other Vesper QTL, *QLr*.*spa-2A*.*2*, was also detected in the Vesper/Stettler population. *QLr*.*spa-2A*.*2* expressed in two out of six Canadian environments and was a moderately strong QTL explaining up to 9% of variation in leaf rust severity and 11% in infection response.

*QLr*.*spa-2B*.*1* was located to the same region in the Carberry/Vesper population as in the Carberry/AC Cadillac population. It was reasonably stable, detected in four of six Canadian environments, and moderately well expressed with explained variation close to 9% in disease severity and 8% in infection response.

*QLr*.*spa-7A*, a relatively weakly expressed QTL, was revealed in two out of six environments. The same resistance QTL was detected, also from Carberry, in the Carberry/AC Cadillac population and from Red Fife in the Stettler/Red Fife population. The third consistent and relatively strongly expressed QTL from Carberry, *QLr*.*spa-7D*, was associated with the *SNP12* marker and was similarly detected in Lillian, but no segregation occurred between Carberry and AC Cadillac at the locus.

### Vesper/Lillian population

Seven leaf rust resistance QTL segregated between Lillian and Vesper; two of them were contributed by Lillian on chromosomes 4A (*QLr*.*spa-4A*) and 7D (*QLr*.*spa-7D*) while five were contributed by Vesper on 1B (*QLr*.*spa-1B*), 1D (*QLr*.*spa-1D*), 6B (*QLr*.*spa-6B*), and 7B (*QLr*.*spa-7B*.*1* and *QLr*.*spa-7B*.*2*) (S1 Fig; Table 4, S6 and S7 Tables). *QLr*.*spa-1B*, although expressed in only a single Canadian environment, explained a reasonable amount the phenotypic variation at 3.7% of the leaf rust severity and 4.9% of the infection response. The peak marker for *QLr*.*spa-1B*, *Wsnp_Ex_c1058_*2020681, was about 19 cM from SSR marker *wmc44* and 3 cM from *gwm328*. The single marker assay and analysis using the *Lr46* marker, *wmc44*, generated a significant marker-trait association. Interestingly, the assay made with the KASP marker associated the *QLr*.*spa-1B* in Vesper produced the same allele among Vesper, Pavon 76, and Lalbahadur.

*QLr*.*spa-1D* was a consistent QTL being expressed in all five test environments which included Canada and New Zealand. It exhibited a major effect explaining phenotypic variation of up to 45% for leaf rust severity and 28% for infection response. Like *QLr*.*spa-1D*, *QLr*.*spa-4A* was also effective in all environments. The QTL spanned a large interval devoid of markers. Associated with SSR markers such as *wmc491* and *wmc680*, the SNP markers *CAP11_c279_66*, *tplb0022j01_1046* and *Ex_c70424_465* were located on one flank of the *QLr*.*spa-4A* QTL (S1 Fig).

*QLr*.*spa-7B*.*2* expressed moderately strongly with close to 10% of the leaf rust severity explained and consistently in four of five environments while *QLr*.*spa-7B*.*1* expressed less strongly and in only a single Canadian environment. The SNP markers within *QLr*.*spa-7B*.*1* were associated with SSR markers *wmc323*, *wmc606*, *gwm537* and *wmc76* whereas *QLr*.*spa-7B*.2 with *gwm146* and *gwm344b* (S1 Fig). *QLr*.*spa-7D* was observed in the same environments as *QLr*.*spa-7B*.*2* which did not include the single year of testing in New Zealand. Maximum expression was greater than that of *QLr*.*spa-7B*.*2*. As previously mentioned, *QLr*.*spa-7D* was associated with *SNP12*.

### Vesper/Stettler population

Two leaf rust resistance QTL, including a major QTL on chromosome 1D (*QLr*.*spa-1D*) and a minor QTL on 2A (*QLr*.*spa-2A*.*2*) were contributed by Vesper (S1 Fig; Table 4, S6 and S7 Tables). However, no QTL was detected from the second parent, Stettler. Similar to the Vesper/Lillian and Carberry/Vesper populations, the *QLr*.*spa-1D* from Vesper/Stettler expressed in all the four tests involving Canada and New Zealand. The QTL explained a considerably large amount of phenotypic variation of up to 45% in leaf rust severity and 28% in infection response. Like in the other two Vesper derived populations, *QLr*.*spa-1D* associated SNP markers mapped close to three SSR markers *wmc432*, *barc149* and *gdm33b* on the consensus map. The QTL on chromosome 2A, *QLr*.*spa-2A*.*2*, although inconsistently detected (two of four environments in Canada) explained substantial phenotypic variation approaching 16% for leaf rust severity and 14% for infection response. The QTL was defined by SNP markers that were located at or near SSR markers *wmc407*, *wmc667*, *gwm296a* and *wmc636b* (S1 Fig).

### Stettler/Red Fife population

Four QTL, one with consistent major effects and three inconsistent with moderate effects on the disease traits, segregated in this population. A QTL on chromosome 2D, *QLr*.*spa-2D*.*2*, and one on 6B, *QLr*.*spa-6B*, were contributed by Stettler. The other two, one on chromosome 7A, *QLr*.*spa-7A*, and one on 7B, *QLr*.*spa-7B*.*2*, were contributed by Red Fife (S1 Fig; Table 4, S6 and S7 Tables). *QLr*.*spa-2D*.*2* consistently reduced leaf rust in Canada and New Zealand with at times strong expression explaining up to 38% of the infection response. This QTL was not detected in a cross of Stettler with Vesper. The other three QTL expressed in single environments at moderate strength: *QLr*.*spa-6B* and *QLr*.*spa-7A* in Canada, and *QLr*.*spa-7B*.*2* in New Zealand. The Stettler 6B QTL had a similar level of expression as the Vesper allele in the Vesper/Lillian population. The two QTL lay in close proximity, with the *Ex_c7101_596* a SNP marker associated with the Stettler QTL located 10 cM from the *BobWhite_c36415_378* marker that flanks the QTL from Vesper. The markers at the peak of the QTL were close to SSR markers *wmc398*, *barc24* and *wmc182a*. Likewise, the *QLr*.*spa-7A* derived from Red Fife was in a similar region to the 7A QTL from Carberry in the Carberry/Vesper and Carberry/AC Cadillac populations. The Red Fife QTL, *QLr*.*spa-7B*.*2*, was located in a similar region to the 7B QTL contributed by AC Cadillac and Vesper.

## Discussion

The results of QTL analysis were consistent with the continuous phenotypic distributions exhibited by the populations. The skewed distributions with a preponderance of leaf rust resistant progenies indicated multiple resistance factors were segregating. Twenty QTL were detected, demonstrating the genetic potential of the five adapted Canadian spring wheat cultivars as sources of leaf rust resistance. In some cases, lack of segregation indicated resistance genes were shared between two parents, for instance *QLr*.*spa-2A*.*2* segregated in Carberry/Vesper and Vesper/Stettler but not in Vesper/Lillian indicating Vesper and Lillian share the same resistance allele. Conversely the resistant allele is not present in Carberry and Stettler. The potential for improved resistance through targeted gene deployment of these QTL comes with minimal to no risk of linkage drag.

The alignment of the QTL maps with the consensus map (S1 Fig) assisted in defining the QTL detected in different varieties and populations. The inclusion of both SSR markers along with SNP markers in our consensus map facilitated the comparisons made between the QTL identified in the present study and previous mapping reports. Other published consensus maps [42, 43] were generated based on SNP markers only.

The majority of the QTL were limited to single parental cultivars as the source of resistance except those located on chromosomes 6B, 7A, 7D and the two on 7B which had two or more cultivars as sources. The discussion from this point on will focus on the QTL as they relate to a specific parental cultivar with associations between cultivars being considered when appropriate.

### QTL detected in a single cultivar

#### Carberry resistance

Carberry was attributed with the highest number of leaf rust resistance QTL in this study. The QTL detected only in Carberry include *QLr*.*spa-1A*, *QLr*.*spa-2B*.*1*, *QLr*.*spa-2B*.*2*, *QLr*.*spa-2D*.*1*, *QLr*.*spa-4B*.*1*, *QLr*.*spa-4B*.*2*, *QLr*.*spa-5A*, and *QLr*.*spa-6A*. Flanked by *IACX1465* and *Excalibur_c46833_204* [42], the 1A QTL, *QLr*.*spa-1A* is located on chromosome 1AS. The spring wheat variety Superb, an immediate parent of Carberry, is known to possess *Lr10* [44] similarly located on 1AS [45]. *Lr10* is one of the widely deployed genes in the western Canadian spring wheat varieties [9, 45–47]. *QLr*.*spa-1A* maps in the same region as the *Lr10* gene, but the relationship between this gene and QTL remains to be determined.

Although our results suggest the two QTL on chromosome 2B from Carberry represent distinct genes, the associated SNP markers place both on the short arm of the chromosome. Flanking markers for *QLr*.*spa-2B*.*1* are located adjacent to two SSR markers, *wmc661* and *wmc764*, which are markers on 2BS for *Lr16* [48, 49]. Consequently *Lr16* is a candidate to explain *QLr*.*spa-2B*.*1*. The second QTL, *QLr*.*spa-2B*.*2*, is next to *wmc154* which corresponds with a complex locus responsible for leaf, stripe and stem rust resistance and expression of pseudo-black chaff [22, 50]. Apart from *Lr16*, Carberry could possess genes such as *Lr13* and *Lr23* similarly located on chromosome 2BS through its immediate parent Alsen [51, 52]. *QLr*.*spa-2B*.*1* and *QLr*.*spa-2B*.*2* were expressed in the majority of tests at a moderate level indicating their usefulness for continued deployment in resistance breeding.

In the present study, we identified two consistent QTL on chromosome 4B, *QLr*.*spa-4B*.*1* and *QLr*.*spa-4B*.*2*, contributed by Carberry. This finding is in partial agreement with Singh et al. [22] who reported only one QTL from Carberry using DArT markers on a subset of the lines from the population we used in the current study. Referring to associated SNP markers *QLr*.*spa-4B*.*1* could be placed on 4BS, and *QLr*.*spa-4B*.*2* on 4BL [42]. A leaf rust resistance locus similar to *QLr*.*spa-4B*.*1* was reported in the Swiss wheat variety Forno [53]. The closest markers for the Carberry QTL were located within 1 cM distance of *gwm368*, a marker for the Forno gene. Chromosome 4BL of wheat harbours genes such as *Lr12* and *Lr31* [54] and *Lr49* [55]; but further work is required to know the true identity of the *QLr*.*spa-4B*.*2* gene.

The SNP marker *BobWhite_c1387_798*, the most highly associated marker with *QLr*.*spa-5A*, the QTL detected from Lillian only, is located on chromosome arm 5AL [42]. To our knowledge there is no designated leaf rust resistance gene reported on this chromosome arm [51]. However, Rosewarne, Singh [56] reported a leaf rust resistance QTL on 5AL in Avocet using a different marker technology making a comparison of relationship difficult. If the QTL in Avocet is different, because no other leaf rust resistance gene has been reported on the long arm of chromosome 5A, *QLr*.*spa-5A* would be novel. The consistency of *QLr*.*spa-5A* makes it valuable in breeding.

#### AC Cadillac resistance

AC Cadillac alone contributed two leaf rust resistance QTL, namely *QLr*.*spa-2A*.*1* and *QLr*.*spa-3B*. The *QLr*.*spa-2A*.*1* mapped in the same region as the QTL that confers multiple disease resistance in Stettler and previously identified in AC Cadillac [22, 24]. Singh, Knox [22] reported the QTL region confers leaf tip necrosis and resistance to leaf rust, stripe rust and powdery mildew. The stripe rust resistance was later confirmed by Bokore, Cuthbert [24]. Like the 2A QTL, the *QLr*.*spa-3B* AC Cadillac leaf rust resistance mapped in the region of stripe rust resistance QTL reported in two different studies [22, 24]. The most highly associated SNP marker to *QLr*.*spa-3B*, *Tdurum_contig79629_538*, mapped close to *UMN10* [41], *Xgwm389* and *csSr2* markers that define the region of the slow rusting stem rust gene *Sr2* [57, 58]. *QLr*.*spa-3B* is in the chromosome region that restricts the development of different fungal diseases with some possibility of relationship to leaf rust resistance gene *Lr27* that is known to be associated with stem rust (*Sr2*) and powdery mildew resistance [59]. Adult plant leaf rust resistance *Lr74* is also located close to *Sr2* in two US winter wheat cultivars Caldwell [60] and Clark [61]. Further investigation is required to know the relationship of the *QLr*.*spa-3B* in Carberry with either *Lr27* or *Lr74*. The multiple disease resistance effects of both *QLr*.*spa-2A*.*1* and *QLr*.*spa-3B* loci make them appealing in breeding.

#### Lillian resistance

Although Lillian was among the most resistant cultivars in the study, only two QTL (*QLr*.*spa-4A* and *QLr*.*spa-7D*) segregated from it in the Vesper/Lillian population. Markers for *QLr*.*spa-4A* were placed with the SSR markers *wmc491* and *wmc680* (S1 Fig) that are assigned to chromosome 4AL [34]. Although the seedling resistance gene *Lr28* is located on 4AL [51, 62], *Lr28* associated SSR markers *wmc313*, *gwm160* and *barc78* [63] are about 105 cM distant from the SNP markers defining *QLr*.*spa-4A* on our map. The only other resistance reported on 4AL was by Gerard et al. [64] but their QTL is located in a similar region as *Lr28/Sr17*. None of the four other cultivars we studied harbored *QLr*.*spa-4A* making Lillian the exception. Perhaps the gene controlling *QLr*.*spa-4A* is less common. Nevertheless it is different from previously reported genes and consequently likely novel. The effectiveness against leaf rust races in Canada and New Zealand of the Lillian *QLr*.*spa-4A* allele make it of great interest for resistance breeding.

#### Stettler resistance

Stettler was the single source of the *QLr*.*spa-2D*.*2* leaf rust resistance and it possessed a second QTL on 6B, *QLr*.*spa-6B* that additionally was detected in Vesper. The *QLr*.*spa-2D*.*2* locus expressed major resistance that was observed in all tests, which spanned the two countries Canada and New Zealand. The *QLr*.*spa-2D*.*2* associated SNP markers were assigned to chromosome 2DS [42] as opposed to the Carberry QTL, *QLr*.*spa-2D*.*1* that is situated on chromosome 2DL based on SNP markers in the QTL interval [42]. on which genes *Lr2a* linked with *gwm484* [65], and *Lr22a* linked with *gwm296*, *gwm455*, and *wmc112* [66] are situated. On our consensus map (S1 Fig), the SNP markers flanking *QLr*.*spa-2D*.*2* place closer to markers for the *Lr2a* than *Lr22a*. Stettler likely inherited *Lr2a* from its immediate parent Superb which is know to possess this gene [44].

#### Vesper resistance

Three QTL located on chromosomes 1B, 1D and 2A were contributed by Vesper. The 1D, *QLr*.*spa-1D* represented a major QTL that consistently expressed in three different populations—Vesper/Lillian, Vesper/Stettler and Carberry/Vesper. The appearance of this QTL in Canada and New Zealand indicated its effectiveness against a diverse spectrum of *P*. *triticina* races. *RAC875_c2070_566* and *BobWhite_c4303_524* that tagged *QLr*.*spa-1D* (S1 Fig) resided on 1DS in the same region with the two SSR markers *gdm33b* and *barc149* that are associated with *Lr21* [67]. Given Vesper is believed to carry *Lr21* through Augusta/Hard White Alpha [68], and *QLr*.*spa-1D* mapped to the short arm of chromosome 1D, it is highly likely to be *Lr21*. Virulence on *Lr21* exists in Canada [11]; however, the results of this study suggest that *Lr21* was still effective in the northern Great Plains, likely due to the relatively low frequency of races virulent to *Lr21* in the inoculum used in this region during the years of testing.

The QTL on 1B (*QLr*.*spa-1B*) coincided with a region for stripe rust resistance previously identified on 1BL in Carberry and Vesper [24]. Markers defining the interval of *QLr*.*spa-1B* were mapped on chromosome 1BL near SSR markers that are associated with the genomic region considered to be pleiotropic for resistance to multiple fungal diseases, *Lr46/Yr29/Pm39/Ltn2* [39, 40]. In addition, *wmc44* linked with *Lr46* [39, 40] displayed significant association with the leaf rust traits in Vesper/Lillian population that indicated *QLr*.*spa-1B* corresponds with *Lr46*.

Markers associated with the 2A Vesper QTL *QLr*.*spa-2A*.*2* discovered in Vesper/Stettler and Carberry/Vesper are located on chromosome 2AS [42]. Many genes have been reported on 2AS including *Lr17a* [69, 70], *Lr37/Yr17/Sr38* [71] and *Lr65* [72]. However, the relatively short distance observed between the SNP markers associated with *QLr*.*spa-2A*.*2* and the SSR marker *wmc407* associated with *Lr17a* [69, 70] make *QLr*.*spa-2A*.*2* the most likely candidate for *Lr17a*.

#### QTL common between cultivars

Out of the twenty QTL identified, QTL that are located on chromosomes 6B, 7A, 7B (2 loci), and 7D were detected in two or more wheat cultivars. For example, the QTL on 6B, *QLr*.*spa-6B* is in common between Vesper and Stettler. Considering the position of the SNP markers detecting the QTL [42] it is located on the long arm of chromosome 6B. Chromosome 6BS is known for having leaf rust resistance genes *Lr36* and *Lr53* [73], whereas, 6BL possesses *Lr3* [74] and *Lr9* [75]. Thus QTL *QLr*.*spa-6B* should be different from *Lr36* and *Lr53* as they are located on different chromosome arms, but it could be *Lr3* or *Lr9*. Virulence to *Lr9* has been developing in Canada most likely starting in 2006 reducing the likelihood that *QLr*.*spa-6B* is Lr9 [76]. There are no reports of the deployment of *Lr3* in the Canadian wheat germplasm making this gene a less likely candidate for *QLr*.*spa-6B*. The uniqueness of gene associated with *QLr*.*spa-6B* will require further study.

*QLr*.*spa-7A* is common between Carberry and Red Fife. The *BS00063860_51* SNP marker associated with the Carberry 7A allele and *tplb0031i24_1212* with the Red Fife 7A allele are 8.0 cM apart, and located on 7AL [42]. A leaf rust resistance gene that was first identified in the Thatcher-*Lr1* near-isogenic Thatcher line RL6003 and temporarily designated as *LrCen* [77] is the only gene recorded on 7AL. The *QLr*.*spa-7A* in the Carberry and Red Fife could be *LrCen*.

The two QTL on 7B, *QLr*.*spa-7B*.*1* and *QLr*.*spa-7B*.*2*, are in common between AC Cadillac and Vesper and additionally Red Fife also has *QLr*.*spa-7B*.2. Markers associated with the *QLr*.*spa-7B*.*1* reside on the short arm of chromosome 7B, while markers for the *QLr*.*spa-7B*.*2* are placed on the long arm of chromosome 7B [42]. No known leaf rust resistance genes are located on the 7BS of bread wheat [51]. However, Herrera-Foessel, Huerta-Espino [78] recently reported the leaf rust resistance gene *Lr72* on the 7BS of durum wheat. Interestingly, *gwm537* one of the markers associated with *Lr72* [78] mapped nearby SNP markers flanking *QLr*.*spa-7B*.*1* (S1 Fig). In contrast, two genes *Lr14a* and *Lr68* are documented as residing on 7BL [51]. The closest marker to the slow rusting gene *Lr68*, *gwm146* [79] was located between about 3.76 to 26.34 cM from SNP markers tagging *QLr*.*spa-7B*.*2* in three different populations (S1 Fig). The SSR marker *gwm146* is also associated with leaf rust resistance QTL in the durum wheat variety Sachem [80]. Herrera-Foessel, Singh [81] reported the seedling resistance gene *Lr14a*, tagged with *gwm146* and *gwm344b*, in a Chilean durum cv. Llareta-INIA. Given *Lr14a* and *Lr68* seem to be closely located genes increases the difficulty attributing one of them to *QLr*.*spa-7B*.*2*.

Carberry and Lillian possess *QLr*.*spa-7D* that corresponded with the *Lr34/Yr18*, a region conferring resistance to leaf, stem and yellow rusts, and powdery mildew [82, 83]. The QTL was consistently detected in Carberry and Lillian by *SNP12*, a diagnostic marker for *Lr34* [27]. *QLr*.*spa-7D* did not segregate in the Carberry/AC Cadillac as both Carberry and AC Cadillac have *Lr34* [84]. The Carberry/AC Cadillac population segregated for 13 QTL; given that Lr34 interacts with other genes to make them more effective [17], it is possible that some of these QTL detected in association with *Lr34* may not be detected in other crosses in which *Lr34* was not fixed with the resistant allele.

In conclusion, the present study showed that although the spring wheat cultivars Lillian, Carberry, AC Cadillac, Stettler, Vesper and Red Fife may have a few leaf rust resistance loci in common, several QTL differ among cultivars that can be further recombined and deployed as gene stacks. The production of the consensus map integrating SNP and SSR markers, enabled us to understand the similarity between the QTL identified in the present mapping study with those leaf rust resistance genes or QTL previously reported based on SSR marker technology. While many of the identified QTL were previously reported rust resistance genes or QTL, others appear to be novel. For example, the resistance genes identified in Lillian at *QLr*.*spa-4A* and Carberry at *QLr*.*spa-5A* could be novel genes. In contrast, the 1D QTL in Vesper corresponded with the designated seedling resistance gene *Lr21*, while the 7D QTL from Lillian and Carberry corresponded with the adult plant resistance gene *Lr34*. The Carberry resistance QTL *QLr*.*spa-2B*.*1* corresponded with *Lr16*, and the 2D QTL from Stettler is more likely *Lr2a* than *Lr22*, but could be another unique gene. Carberry demonstrated substantial stacking of genes which could be supplemented with the genes identified in the other adapted varieties with the expectation of increasing efficacy and longevity of resistance to leaf rust with little risk of linkage drag in Canadian wheat breeding programs. Some of the SNP markers associated with the identified QTL have been converted to KASP markers that are being deployed in germplasm evaluation and breeding for marker assisted stacking of leaf rust resistance to develop new varieties.

## Supporting information

S1 File Hexaploid wheat consensus map and genetic maps of the 14 different populations used to build the consensus map. (XLSX)

S1 Table Pedigree description and expected leaf rust resistance genes of wheat cultivars used in to generate five mapping populations. (DOCX)

S2 Table Number of lines tested for leaf rust severity and infection response and genotyped by SNP markers in five doubled haploid populations, years of evaluation and location of field nurseries in Canada (Brandon, Morden and Swift Current) and New Zealand (Lincoln). (DOCX)

S3 Table Roche LightCycler II High Resolution Melt (HRM) Programming for SNP12 marker assay. (DOCX)

S4 Table Leaf rust severity (%) and infection responses of five doubled haploid populations evaluated along with population parents in nurseries near Morden and Brandon, MB, and Swift Current, SK, Canada and near Lincoln, New Zealand. (DOCX)

S5 Table Summary statistics of the individual maps of the 14 hexaploid wheat populations used to generate the consensus hexaploid wheat map. (DOCX)

S6 Table The number of environments in which each of five mapping populations was field evaluated for adult plant leaf rust resistance, the test environments in which QTL were identified and the parental source of the resistance allele (in parentheses). (DOCX)

S7 Table Closest marker, associated LOD score, mean phenotypic values associated with parental molecular variants, percentage of phenotypic variation explained by individual QTL and the level of additive effect of the QTL detected in five doubled haploid populations evaluated for field responses against leaf rust in nurseries near Swift Current, SK, and Morden, MB, Canada and Lincoln, New Zealand. (DOCX)

S8 Table Location of the leaf rust resistance QTL associated markers in the International Wheat Genome Sequencing Consortium (IWGSC) RefSeq. V. 1.0 wheat genome assembly. (DOCX)

S1 Fig Partial genetic linkage map of quantitative trait loci for resistance to leaf rust in five wheat populations aligned with the hexaploid wheat consensus map constructed using SSR and SNP markers. Some markers were removed from the linkage maps illustrated here for simplification of the presentation. The test environments are denoted by the test years preceded by an abbreviation of the location as follows: MD, Morden; BD, Brandon; SC, Swift Current, Canada, and LN, Lincoln, New Zealand. The disease phenotypes are abbreviated: LRSEV, leaf rust severity, and LRIR, leaf rust infection response. Chromosome names are followed by population name abbreviations: CCd, Carberry/AC Cadillac; CV, Carberry/Vesper; VL, Vesper/Lillian; VS, Vesper/Stettler; RS, Red Fife/Stettler.
(DOCX)
