# Supplementary material for: Mapping quantitative trait loci associated with leaf rust resistance in five spring wheat populations using single nucleotide polymorphism markers
Source: PLoS One. 2020 Apr 8;15(4):e0230855. doi: 10.1371/journal.pone.0230855 (PMC7141615; doi:10.1371/journal.pone.0230855)
Supplement: S1 Table — (DOCX) [file pone.0230855.s002.docx]

**S1 Table. Pedigree description and possible leaf rust resistance genes of wheat cultivars used as parents to generate five mapping populations.**

| **Cultivar name** | **Pedigree** | **Expected leaf rust resistance genes** |
| --- | --- | --- |
| AC Cadillac | BW90*3/BW553 | *Lr34* (Hiebert et al. 2011) |
| Carberry | Alsen/Superb | *Lr2a* (2DS) and *Lr10* (1AS) from Superb (McCallum and Seto-Goh 2010). Seedling genes *Lr2a*, *Lr10*, and *Lr23* (2BS) and adult plant genes *Lr13* (2BS) and *Lr34* (7DS) from Alsen (Oelke and Kolmer 2005) |
| Lillian | BW621*3/90B07-AU2B;  BW621 = Leader/7504-78//7504-59; 90B07-AU2B = Pasqua*2/ND643 | Lillian inherited *Lr34* through BW621 or Pasqua (McCallum et al. 2012) |
| Stettler | Prodigy/Superb; Prodigy = Columbus/BW85//Stoa; BW 85 = Neepawa*5/Buck Manantial | *Lr2a* and *Lr10* from Superb (McCallum and Seto-Goh 2010) |
| Vesper | Augusta/Hard White Alpha//3*AC Barrie/6/BW150*2//Tp/Tm/3/2*Superb/4/94B35-R5C/5/Superb | *Lr21* from Augusta/Hard White Alpha (Thomas et al. 2013) |
