# Supplementary material for: Mapping quantitative trait loci associated with leaf rust resistance in five spring wheat populations using single nucleotide polymorphism markers
Source: PLoS One. 2020 Apr 8;15(4):e0230855. doi: 10.1371/journal.pone.0230855 (PMC7141615; doi:10.1371/journal.pone.0230855)
Supplement: S2 Table — (DOCX) [file pone.0230855.s003.docx]

**S2 Table. Number of lines tested for leaf rust severity and infection response and genotyped by SNP markers in five doubled haploid populations, years of evaluation and location of field nurseries in Canada (Brandon, Morden and Swift Current) and New Zealand (Lincoln).**

| **Population** | **Number of lines genotyped** | **Swift Current** | | | | | **Morden** | | | **Brandon** | **Lincoln** |
| --- | --- | --- | --- | --- | --- | --- | --- | --- | --- | --- | --- |
|  |  | **2011 ^a^** | **2012** | **2013** | **2014** | **2015** | **2015** | **2016** | **2017** | **2016** | **2014** |
| Carberry/AC Cadillac | 775 | 812 ^b^ | 812 | 812 | 232 |  |  |  |  |  |  |
| Carberry/Vesper | 188 |  |  |  | 253 | 180 | 180 | 180 | 180 | 180 | 188^c^ |
| Vesper/Lillian | 283 |  |  | 328 | 360 | 282 | 282 |  |  |  | 282 |
| Vesper/Stettler | 94 |  |  |  | 194 | 94 | 94 |  |  |  | 94 |
| Stettler/Red Fife | 218 |  |  |  | 132 | 226 | 226 |  |  |  | 226 |

^a^ Year of field evaluation

^b^ Number of DH lines evaluated in each nursery

^c^ Data of Carberry/Vesper from Lincoln was not used in QTL analysis due to low disease values and poor differentiation between lines
