# Supplementary material for: Mapping quantitative trait loci associated with leaf rust resistance in five spring wheat populations using single nucleotide polymorphism markers
Source: PLoS One. 2020 Apr 8;15(4):e0230855. doi: 10.1371/journal.pone.0230855 (PMC7141615; doi:10.1371/journal.pone.0230855)
Supplement: S3 Table — (DOCX) [file pone.0230855.s004.docx]

**S3 Table. Roche LightCycler II High Resolution Melt (HRM) Programming for SNP12 marker assay.**

| **Program Name** | **Cycles** | **Temperature (°C)** | **Hold Time** | **Ramp Rate (°C/s)** | **Acquisitions (per °C)** | **Acquisition mode** | **Analysis Mode** |
| --- | --- | --- | --- | --- | --- | --- | --- |
| Pre-incubation | 1 | 95 | 10 min | 4.4 | --- | None | None |
| Denaturation | 45 | 95 | 10 sec | 4.4 | --- | None | Quantification |
| Anneal | 45 | 2 °C below I° Tm | 15 sec | 2.2 | --- | None | Quantification |
| Extension | 45 | 72 | 20 sec | 4.4 | --- | Single | Quantification |
| HRM 95 °C | 1 | 95 | 60 sec | 4.4 | --- | None | Melting Curve |
| HRM 40 °C | 1 | 40 | 60 sec | 2.2 | --- | None | Melting Curve |
| HRM 65 °C | 1 | 65 | 1 sec | 1 | --- | None | Melting Curve |
| HRM 95 °C | 1 | 95 | --- | --- | 25 | Continuous | Melting Curve |
| Cooling 40 °C | 1 | 40 | 10 sec | 2.2 | --- | None | None |
