# Supplementary material for: Mapping quantitative trait loci associated with leaf rust resistance in five spring wheat populations using single nucleotide polymorphism markers
Source: PLoS One. 2020 Apr 8;15(4):e0230855. doi: 10.1371/journal.pone.0230855 (PMC7141615; doi:10.1371/journal.pone.0230855)
Supplement: S4 Table — (DOCX) [file pone.0230855.s005.docx]

**S4 Table. Leaf rust severity (%) and infection responses of five doubled haploid populations evaluated along with population parents in nurseries near Morden and Brandon, MB, and Swift Current, SK, Canada and near Lincoln, New Zealand.**

| Population/Environment |  | Swift Current | | | | |  | Morden | | |  | Brandon |  | Lincoln |
| --- | --- | --- | --- | --- | --- | --- | --- | --- | --- | --- | --- | --- | --- | --- |
|  |  | 2011^a^ | 2012 | 2013 | 2014 | 2015 |  | 2015 | 2016 | 2017 |  | 2016 |  | 2014 |
| Carberry/AC Cadillac | Carberry | 12.5RMR | 2.7RMR | 2.6MR | 5RMR |  |  |  |  |  |  |  |  |  |
|  | AC Cadillac | 31.7MR | 20MR | 21.2MR | 4.5RMR |  |  |  |  |  |  |  |  |  |
|  | Range | 0.5-80 | 0.5-70 | 0.5-80 | 1-40 |  |  |  |  |  |  |  |  |  |
|  | Mean | 26.2 | 13.5 | 13.5 | 8.0 |  |  |  |  |  |  |  |  |  |
| Carberry/Vesper | Carberry |  |  |  | 3MR | 12.8RMR |  | 15MR | 3R | 3.5RMR |  | TrR |  | 0 |
|  | Vesper |  |  |  | 3MR | 5RMR |  | 30MRMS | 5RMR | 12.5MRMS |  | 11.5MRMS |  | 0 |
|  | Range |  |  |  | 0.5-30 | 0.5-50 |  | 0-80 | 0.5-60 | 0-40 |  | 0.5-35 |  | 0-40 |
|  | Mean |  |  |  | 4.1 | 10.7 |  | 25.7 | 8.8 | 7.3 |  | 6.8 |  | 1 |
| Vesper/Lillian | Lillian |  |  |  | 2.7MR | 10.0MR |  | 2.8RMR |  |  |  |  |  | 0 |
|  | Vesper |  |  |  | 0.7MR | TrR |  | 15MR |  |  |  |  |  | 0 |
|  | Range |  |  | 1-50 | 0-60 | 1-55 |  | 1-95 |  |  |  |  |  | 0-80 |
|  | Mean |  |  | 18.7 | 6.9 | 8 |  | 30.4 |  |  |  |  |  | 15 |
| Vesper/Stettler | Stettler |  |  |  | 5MR | 10MR |  | 60S |  |  |  |  |  | 0 |
|  | Vesper |  |  |  | 0.5R | 7.5MR |  | 17.5MRMS |  |  |  |  |  | 0 |
|  | Range |  |  |  | 1-45 | 1-50 |  | 1-95 |  |  |  |  |  | 0-60 |
|  | Mean |  |  |  | 4.2 | 21.1 |  | 36.8 |  |  |  |  |  | 5 |
| Stettler/Red Fife | Red Fife |  |  |  | 7MR | 17.5MR |  | 77.5S |  |  |  |  |  | 30 |
|  | Stettler |  |  |  | 10MR | 3.5MR |  | 45MRMS |  |  |  |  |  | 0 |
|  | Range |  |  |  | 2-20 | 0.5-50 |  | 0.5-95 |  |  |  |  |  | 0-60 |
|  | Mean |  |  |  | 7.9 | 8.7 |  | 39.4 |  |  |  |  |  | 6 |

^a^ Infection response: R, resistant; RMR, resistant to moderately resistant; MR, moderately resistant; MRMS, moderately resistant to moderately susceptible; MS, moderately susceptible; MSS, moderately susceptible to susceptible; and S, susceptible
