# Supplementary material for: Mapping quantitative trait loci associated with leaf rust resistance in five spring wheat populations using single nucleotide polymorphism markers
Source: PLoS One. 2020 Apr 8;15(4):e0230855. doi: 10.1371/journal.pone.0230855 (PMC7141615; doi:10.1371/journal.pone.0230855)
Supplement: S5 Table — (DOCX) [file pone.0230855.s006.docx]

**S5 Table. Summary statistics of the individual maps of the 14 hexaploid wheat populations used to generate the consensus hexaploid wheat map.**

| **Population** | **Number of markers** | **Map length (cM)** | **Marker density (cM/marker)** |
| --- | --- | --- | --- |
| 8021V2/AC Karma | 13263 | 3890.2 | 0.3 |
| AAC Concord/CDC Hughes | 9648 | 2920.1 | 0.3 |
| Attila/CDC Go | 5030 | 2537.0 | 0.5 |
| Carberry/AC Cadillac | 6806 | 4527.6 | 0.7 |
| Carberry/Thatcher | 6914 | 3779.0 | 0.5 |
| Carberry/Vesper | 6138 | 1835.4 | 0.3 |
| Cutler/AC Barrie | 10359 | 5077.2 | 0.8 |
| Norstar/Winter Manitou | 9495 | 2860.9 | 0.3 |
| Norstar/Capelle Desprez | 10487 | 3321.6 | 0.3 |
| Norstar/Manitou | 8431 | 3045.9 | 0.4 |
| RL4452/AC Domain | 11160 | 2626.8 | 0.2 |
| Stettler/Red Fife | 9983 | 3247.6 | 0.3 |
| Vesper/Lillian | 8040 | 4099.6 | 0.8 |
| Vesper/Stettler | 4989 | 2002.0 | 0.4 |
