# Supplementary material for: Mapping quantitative trait loci associated with leaf rust resistance in five spring wheat populations using single nucleotide polymorphism markers
Source: PLoS One. 2020 Apr 8;15(4):e0230855. doi: 10.1371/journal.pone.0230855 (PMC7141615; doi:10.1371/journal.pone.0230855)
Supplement: S6 Table — (DOCX) [file pone.0230855.s007.docx]

**S6 Table. The number of environments in which each of five mapping populations was field evaluated for adult plant leaf rust resistance, the test environments in which QTL were identified and the parental source of the resistance allele (in parentheses).**

| **Chromosome** | **QTL** | **Possible gene** | **Carberry/AC Cadillac** | **Carberry/Vesper** | **Vesper/Lillian** | **Vesper/Stettler** | **Stettler/Red Fife** |
| --- | --- | --- | --- | --- | --- | --- | --- |
|  |  |  | **4 ^a^** | **6** | **5** | **4** | **4** |
| 1A | *QLr.spa-1A* | *Lr10* | SC11, SC12, SC13 (C) ^b, c^ |  |  |  |  |
| 1B | *QLr.spa-1B* | *Lr46* |  |  | SC2014 (V) |  |  |
| 1D | *QLr.spa-1D* | *Lr21* |  | SC14, SC15, MD15, BD16 (V) | SC13, SC14, SC15, LN14, MD15 (V) | SC14, SC15, MD15, LN14 (V) |  |
| 2A | *QLr.spa-2A.1* |  | SC11, SC12, SC13 (Cd) |  |  |  |  |
| 2A | *QLr.spa-2A.2* | *Lr17a* |  | MD15, BD16 (V) |  | SC14, MD15 (V) |  |
| 2B | *QLr.spa-2B.1* | *Lr16* | SC11, SC12 (C) | MD15, MD16, MD17, BD16 (C) |  |  |  |
| 2B | *QLr.spa-2B.2* |  | SC11, SC12, SC13 (C) |  |  |  |  |
| 2D | *QLr.spa-2D.1* |  | SC11, SC12 (C) |  |  |  |  |
| 2D | *QLr.spa-2D.2* | *Lr2a* |  |  |  |  | SC14, SC15, MD15, LN14 (S) |
| 3B | *QLr.spa-3B* | *Lr27* | SC2011, SC2013 (Cd) |  |  |  |  |
| 4A | *QLr.spa-4A* |  |  |  | SC13, SC14, SC15, MD15, LN14 (L) |  |  |
| 4B | *QLr.spa-4B.1* |  | SC11, SC12, SC13 (C) |  |  |  |  |
| 4B | *QLr.spa-4B.2* |  | SC11, SC12, SC13 (C) |  |  |  |  |
| 5A | *QLr.spa-5A* |  | SC11, SC12, SC13, SC14 (C) |  |  |  |  |
| 6A | *QLr.spa-6A* |  | SC12, SC13 (C) |  |  |  |  |
| 6B | *QLr.spa-6B* |  |  |  | SC13, SC14, LN14 (V) |  | SC15 (S) |
| 7A | *QLr.spa-7A* | *LrCen* | SC13 (C) | SC2014, SC2015 (C) |  |  | MD15 (R) |
| 7B | *QLr.spa-7B.1* | *Lr72* | SC11, SC12, SC13, SC14 (Cd) |  | SC13 (V) |  |  |
| 7B | *QLr.spa-7B.2* | *Lr14a* or *Lr68* | SC11, SC12, SC13 (Cd) |  | SC13, SC14, SC15, MD15 (V) |  | LN14 (R) |
| 7D | *QLr.spa-7D* | *Lr34* |  | SC14, SC15, MD15, MD16, MD17, BD16 (C) | SC13, SC14, SC15, MD15 (L) |  |  |

^a^ Total number of test environments per population

^b^ Location names followed by the last two digits of test years: SC, Swift Current; BD, Brandon; and MD, Morden, Canada; LN, Lincoln, New Zealand

^c^ Letter abbreviations in parentheses indicate the source of leaf rust resistance alleles: Cd, AC Cadillac; C, Carberry; L, Lillian; R, Red Fife; S, Stettler; V, Vesper.
