# Supplementary material for: Mapping quantitative trait loci associated with leaf rust resistance in five spring wheat populations using single nucleotide polymorphism markers
Source: PLoS One. 2020 Apr 8;15(4):e0230855. doi: 10.1371/journal.pone.0230855 (PMC7141615; doi:10.1371/journal.pone.0230855)
Supplement: S7 Table — (DOCX) [file pone.0230855.s008.docx]

**S7 Table. Closest marker, associated LOD score, mean phenotypic values associated with parental molecular variants, percentage of phenotypic variation explained by individual QTL and the level of additive effect of the QTL detected in five doubled haploid populations evaluated for field responses against leaf rust in nurseries near Swift Current, SK, and Morden, MB, Canada and Lincoln, New Zealand.**

| **Population^a^** | **Chromosome** | **QTL** | **Trait^b^** | **Location-Year^c^** | **Marker** | **Pos., cM** | **LOD^d^** | **Mean of AC Cadillac allele** | **Mean of Carberry allele** | **PVE%^e^** | **Additive effect** | **Source of resistance allele** |
| --- | --- | --- | --- | --- | --- | --- | --- | --- | --- | --- | --- | --- |
| C/Cd | 1A | *QLr.spa-1A* | S | SC2011 | IACX1465 | 42.689 | 8.4 | 31.7 | 12.6 | 4.8 | 9.6 | C |
| C/Cd | 1A | *QLr.spa-1A* | IR | SC2011 | IACX1465 | 42.689 | 3.4 | 3.6 | 2.4 | 2 | 0.6 | C |
| C/Cd | 1A | *QLr.spa-1A* | S | SC2012 | IACX1465 | 42.689 | 15.1 | 18.2 | 3.7 | 8.6 | 7.3 | C |
| C/Cd | 1A | *QLr.spa-1A* | IR | SC2012 | Excalibur_c46833_204 | 45.086 | 3.8 | 4.8 | 3.8 | 2.2 | 0.5 | C |
| C/Cd | 1A | *QLr.spa-1A* | S | SC2013 | IACX1465 | 42.689 | 11.6 | 17.2 | 3 | 6.7 | 7.1 | C |
| C/Cd | 1A | *QLr.spa-1A* | IR | SC2013 | Excalibur_c46833_204 | 45.086 | 4 | 3.1 | 2.4 | 2.3 | 0.4 | C |
| C/Cd | 2A | *QLr.spa-2A.1* | S | SC2011 | BS00041816_51 | 52.631 | 4.6 | 19.5 | 25.4 | 2.7 | -2.9 | Cd |
| C/Cd | 2A | *QLr.spa-2A.1* | IR | SC2011 | BS00022241_51 | 48.876 | 10.2 | 2.5 | 3.1 | 1.8 | -0.3 | Cd |
| C/Cd | 2A | *QLr.spa-2A.1* | S | SC2012 | BS00022241_51 | 49.876 | 4.2 | 9.6 | 12.8 | 2.4 | -1.6 | Cd |
| C/Cd | 2A | *QLr.spa-2A.1* | IR | SC2012 | BS00022241_51 | 49.876 | 10.1 | 3.1 | 3.5 | 5.8 | -0.2 | Cd |
| C/Cd | 2A | *QLr.spa-2A.1* | S | SC2013 | BS00022241_51 | 49.876 | 7.06 | 8.1 | 12.7 | 4.1 | -2.3 | Cd |
| C/Cd | 2A | *QLr.spa-2A.1* | IR | SC2013 | BS00022241_51 | 48.876 | 7.2 | 2.5 | 3 | 4.2 | -0.3 | Cd |
| C/Cd | 2B | *QLr.spa-2B.1* | S | SC2011 | Excalibur_c39493_251 | 7.443 | 3.38 | 27.4 | 17.9 | 2.0 | 4.7 | C |
| C/Cd | 2B | *QLr.spa-2B.1* | IR | SC2012 | BobWhite_c12144_216 | 0 | 3.31 | 4.6 | 4.0 | 1.9 | 0.3 | C |
| C/Cd | 2B | *QLr.spa-2B.2* | S | SC2011 | *Kukri_c53810_137* | 38.8 | 6.4 | 30.5 | 13.8 | 3.6 | 8.3 | C |
| C/Cd | 2B | *QLr.spa-2B.2* | IR | SC2011 | *Kukri_c53810_137* | 38.8 | 3.5 | 3.6 | 2.4 | 2 | 0.6 | C |
| C/Cd | 2B | *QLr.spa-2B.2* | S | SC2012 | *Kukri_c53810_137* | 38.8 | 10.6 | 17 | 4.8 | 5.9 | 6.1 | C |
| C/Cd | 2B | *QLr.spa-2B.2* | IR | SC2012 | *Kukri_c53810_137* | 38.8 | 3.8 | 3.5 | 3.2 | 2.2 | 0.2 | C |
| C/Cd | 2B | *QLr.spa-2B.2* | S | SC2013 | *Kukri_c53810_137* | 38.8 | 15.5 | 18.2 | 1.8 | 8.7 | 8.2 | C |
| C/Cd | 2B | *QLr.spa-2B.2* | IR | SC2013 | *Kukri_c53810_137* | 38.8 | 3.3 | 3.2 | 2.3 | 1.8 | 0.4 | C |
| C/Cd | 2D | *QLr.spa-2D.1* | S | SC2011 | *Ex_c2115_3369* | 81.7 | 3.9 | 25.4 | 20 | 2.3 | 2.7 | C |
| C/Cd | 2D | *QLr.spa-2D.1* | S | SC2012 | *RAC875_c2092_1020* | 91.6 | 3.4 | 12.8 | 9.9 | 2 | 1.5 | C |
| C/Cd | 3B | QLr.spa-3B | S | SC2011 | *Tdurum_contig79629_538* | 15.062 | 2.07 | 20.6 | 24.6 | 1.2 | -2 | Cd |
| C/Cd | 3B | QLr.spa-3B | IR | SC2011 | *Tdurum_contig79629_538* | 15.062 | 2.57 | 4.1 | 4.5 | 1.5 | -0.2 | Cd |
| C/Cd | 3B | QLr.spa-3B | S | SC2013 | *Tdurum_contig79629_538* | 15.062 | 3.99 | 8.8 | 12.3 | 2.3 | -1.7 | Cd |
| C/Cd | 3B | QLr.spa-3B | IR | SC2013 | *Tdurum_contig79629_538* | 15.062 | 4.78 | 2.6 | 3 | 2.8 | -0.2 | Cd |
| C/Cd | 4B | *QLr.spa-4B.1* | S | SC2011 | *Tdurum_contig12204_1131* | 0 | 3.4 | 25 | 20 | 1.9 | 2.5 | C |
| C/Cd | 4B | *QLr.spa-4B.1* | IR | SC2011 | *Tdurum_contig12204_1131* | 0 | 3 | 3.2 | 2.8 | 1.7 | 0.2 | C |
| C/Cd | 4B | *QLr.spa-4B.1* | S | SC2012 | *Tdurum_contig12204_1131* | 0 | 3.3 | 12.7 | 9.8 | 1.9 | 1.4 | C |
| C/Cd | 4B | *QLr.spa-4B.1* | IR | SC2012 | *Tdurum_contig12204_1131* | 0 | 5.3 | 4.6 | 4 | 3 | 0.3 | C |
| C/Cd | 4B | *QLr.spa-4B.1* | S | SC2013 | *Tdurum_contig12204_1131* | 0 | 3.5 | 12.1 | 8.8 | 2 | 1.6 | C |
| C/Cd | 4B | *QLr.spa-4B.2* | S | SC2011 | *BS00021984_51* | 75.7 | 4.5 | 25.4 | 19.6 | 2.6 | 2.9 | C |
| C/Cd | 4B | *QLr.spa-4B.2* | IR | SC2011 | *BS00021984_51* | 75.7 | 5.2 | 3.3 | 2.7 | 3 | 0.3 | C |
| C/Cd | 4B | *QLr.spa-4B.2* | S | SC2012 | *Ex_c101685_705* | 78.6 | 3.3 | 12.7 | 9.9 | 1.9 | 1.4 | C |
| C/Cd | 4B | *QLr.spa-4B.2* | IR | SC2012 | *BS00021984_51* | 75.7 | 6.3 | 4.7 | 4 | 3.6 | 0.3 | C |
| C/Cd | 4B | *QLr.spa-4B.2* | S | SC2013 | *BS00021984_51* | 75.7 | 3 | 11.9 | 9 | 1.7 | 1.5 | C |
| C/Cd | 4B | *QLr.spa-4B.2* | IR | SC2013 | *Ex_c101685_705* | 78.6 | 6.7 | 3 | 2.5 | 3.8 | 0.3 | C |
| C/Cd | 5A | *QLr.spa-5A* | S | SC2011 | *BobWhite_c1387_798* | 41.634 | 4.5 | 25.4 | 19.3 | 2.6 | 3.1 | C |
| C/Cd | 5A | *QLr.spa-5A* | IR | SC2011 | *BobWhite_c1387_798* | 41.634 | 4.8 | 3 | 2.6 | 2.8 | 0.2 | C |
| C/Cd | 5A | *QLr.spa-5A* | S | SC2012 | *BobWhite_c1387_798* | 41.634 | 7.9 | 13.5 | 8.9 | 4.6 | 2.3 | C |
| C/Cd | 5A | *QLr.spa-5A* | S | SC2013 | *BobWhite_c1387_798* | 41.634 | 3.1 | 3.4 | 3.2 | 1.8 | 0.1 | C |
| C/Cd | 5A | *QLr.spa-5A* | IR | SC2013 | *BobWhite_c1387_798* | 41.634 | 9.4 | 13.1 | 7.6 | 5.4 | 2.8 | C |
| C/Cd | 5A | *QLr.spa-5A* | S | SC2014 | *Kukri_c13590_344* | 41.3 | 3.8 | 9.8 | 6.3 | 7.3 | 1.8 | C |
| C/Cd | 6A | *QLr.spa-6A* | S | SC2012 | *BobWhite_c39821_195* | 4.19 | 11.5 | 17.1 | 4.4 | 6.6 | 6.4 | C |
| C/Cd | 6A | *QLr.spa-6A* | S | SC2013 | *BobWhite_c39821_195* | 4.19 | 14.7 | 17.8 | 1.9 | 8.3 | 8 | C |
| C/Cd | 7A | *QLr.spa-7A* | S | SC2013 | *BS00063860_51* | 193.3 | 3.8 | 12.2 | 8.8 | 2.2 | 1.7 | C |
| C/Cd | 7B | *QLr.spa-7B.1* | S | SC2011 | *Ex_c101666_634* | 31.5 | 9.2 | 18.2 | 26.4 | 5.2 | -4.1 | Cd |
| C/Cd | 7B | *QLr.spa-7B.1* | IR | SC2011 | *Ex_c101666_634* | 31.5 | 12.4 | 2.5 | 3.5 | 7 | -0.5 | Cd |
| C/Cd | 7B | *QLr.spa-7B.1* | S | SC2012 | *Ex_c101666_634* | 31.5 | 15. 5 | 8.1 | 14.1 | 8.6 | -3 | Cd |
| C/Cd | 7B | *QLr.spa-7B.1* | IR | SC2012 | *Ex_c101666_634* | 31.5 | 14.9 | 3 | 3.6 | 8.5 | -0.3 | Cd |
| C/Cd | 7B | *QLr.spa-7B.1* | S | SC2013 | *Ex_c101666_634* | 31.5 | 18 | 6.7 | 13.9 | 9.9 | -3.6 | Cd |
| C/Cd | 7B | *QLr.spa-7B.1* | IR | SC2013 | *Ex_c101666_634* | 31.5 | 18.2 | 2.3 | 3.2 | 10.2 | -0.4 | Cd |
| C/Cd | 7B | *QLr.spa-7B.1* | S | SC2014 | *Ex_c101666_634* | 31.5 | 2. 8 | 5.96 | 8.76 | 4.5 | -1.4 | Cd |
| C/Cd | 7B | *QLr.spa-7B.1* | IR | SC2014 | *Ex_c101666_634* | 31.5 | 5.8 | 2.8 | 3.57 | 10.7 | -0.4 | Cd |
| C/Cd | 7B | *QLr.spa-7B.2* | S | SC2011 | RAC875_c57326_85 | 145.9 | 4. 5 | 14.4 | 29.3 | 2.4 | -7.4 | Cd |
| C/Cd | 7B | *QLr.spa-7B.2* | S | SC2012 | RAC875_c57326_85 | 145.9 | 21.3 | 1.7 | 19.4 | 10.6 | -8.8 | Cd |
| C/Cd | 7B | *QLr.spa-7B.2* | S | SC2013 | RAC875_c57326_85 | 145.9 | 15.2 | 1.5 | 18.1 | 7.6 | -8.3 | Cd |
| C/Cd | 7B | *QLr.spa-7B.2* | IR | SC2013 | RAC875_c57326_85 | 145.9 | 3 | 2.3 | 3.1 | 1.6 | -0.4 | Cd |
| **Population** | **Chromosome** | **QTL** | **Trait** | **Location-Year** | **Marker** | **Pos., cM** | **LOD** | **Mean of Carberry allele** | **Mean of Vesper allele** | **PVE,%** | **Additive effect** | **Source of resistance allele** |
| C/V | 1D | *QLr.spa-1D* | S | SC2014 | *RAC875_c2070_566* | 67.5 | 6 | 5.8 | 2.5 | 13.8 | 1.6 | V |
| C/V | 1D | *QLr.spa-1D* | IR | SC2014 | *RAC875_c2070_566* | 67.5 | 5 | 3.6 | 2.8 | 11.6 | 0.4 | V |
| C/V | 1D | *QLr.spa-1D* | S | SC2015 | *RAC875_c2070_566* | 67.5 | 6.7 | 15.1 | 6.1 | 15.7 | 4.5 | V |
| C/V | 1D | *QLr.spa-1D* | IR | SC2015 | *RAC875_c2070_566* | 67.5 | 5.5 | 4.5 | 1.8 | 11.8 | 1.3 | V |
| C/V | 1D | *QLr.spa-1D* | S | MD2015 | *RAC875_c2070_566* | 67.5 | 9.7 | 36.4 | 14.5 | 21.9 | 11 | V |
| C/V | 1D | *QLr.spa-1D* | IR | MD2015 | *RAC875_c2070_566* | 67.5 | 8.4 | 6.2 | 3.8 | 19.4 | 1.2 | V |
| C/V | 1D | *QLr.spa-1D* | S | BD2016 | *RAC875_c2070_566* | 67.5 | 2.9 | 8.6 | 4.9 | 7.1 | 1.9 | V |
| C/V | 2A | *QLr.spa-2A.2* | S | BD2016 | *Kukri_c46040_620* | 0 | 3.6 | 8.8 | 4.6 | 8.8 | 2.1 | V |
| C/V | 2A | *QLr.spa-2A.2* | IR | MD2015 | *Kukri_c46040_620* | 0 | 4.7 | 5.9 | 4 | 11.3 | 0.9 | V |
| C/V | 2B | *QLr.spa-2B.1* | S | MD2015 | *BobWhite_c12144_216* | 0 | 2.5 | 20 | 31.6 | 6.1 | -5.8 | C |
| C/V | 2B | *QLr.spa-2B.1* | S | BD2016 | *BobWhite_c12144_216* | 0 | 2.5 | 5.1 | 8.6 | 6.3 | -1.7 | C |
| C/V | 2B | *QLr.spa-2B.1* | S | MD2016 | *BobWhite_c12144_216* | 0 | 2.8 | 6.1 | 11.7 | 6.8 | -2.8 | C |
| C/V | 2B | *QLr.spa-2B.1* | IR | MD2016 | *BobWhite_c12144_216* | 0 | 2.7 | 2.2 | 3 | 6.7 | -0.4 | C |
| C/V | 2B | *QLr.spa-2B.1* | S | MD2017 | *BobWhite_c12144_216* | 0 | 3.6 | 5.1 | 9.5 | 8.8 | -2.2 | C |
| C/V | 2B | *QLr.spa-2B.1* | IR | MD2017 | *BobWhite_c12144_216* | 0 | 3.3 | 2.3 | 3.2 | 8.2 | -0.5 | C |
| C/V | 7A | *QLr.spa-7A* | S | SC2014 | *BS00053365_51* | 2.43 | 3 | 5.2 | 5.8 | 2.4 | -1.1 | C |
| C/V | 7A | *QLr.spa-7A* | S | SC2015 | *BS00053365_51* | 3.02 | 7.6 | 13.8 | 7.4 | 3 | -3.1 | C |
| C/V | 7A | *QLr.spa-7A* | IR | SC2015 | *BS00053365_51* | 2.61 | 2.6 | 3.9 | 6.5 | 2.6 | -0.7 | C |
| C/V | 7D | *QLr.spa-7D* | IR | SC2014 | *SNP12* | 2 | 3.3 | 2.8 | 3.5 | 7.9 | -0.3 | C |
| C/V | 7D | *QLr.spa-7D* | IR | SC2015 | *SNP12* | 2 | 3.6 | 2.4 | 3.9 | 8.7 | -0.8 | C |
| C/V | 7D | *QLr.spa-7D* | S | MD2015 | *SNP12* | 2 | 7.5 | 15.7 | 35.3 | 17.5 | -9.8 | C |
| C/V | 7D | *QLr.spa-7D* | IR | MD2015 | *SNP12* | 2 | 6.5 | 3.9 | 6.1 | 15.4 | -1.1 | C |
| C/V | 7D | *QLr.spa-7D* | S | BD2016 | *SNP12* | 2 | 8 | 3.7 | 9.7 | 18.5 | -3 | C |
| C/V | 7D | *QLr.spa-7D* | IR | BD2016 | *SNP12* | 2 | 7 | 1.8 | 3 | 16.5 | -0.6 | C |
| C/V | 7D | *QLr.spa-7D* | S | MD2016 | *SNP12* | 2 | 5.9 | 4.7 | 12.7 | 14 | -4 | C |
| C/V | 7D | *QLr.spa-7D* | IR | MD2016 | *SNP12* | 2 | 5.4 | 2 | 3.1 | 12.9 | -0.6 | C |
| C/V | 7D | *QLr.spa-7D* | S | MD2017 | *SNP12* | 2 | 9.9 | 3.6 | 10.7 | 22.4 | -3.5 | C |
| C/V | 7D | *QLr.spa-7D* | IR | MD2017 | *SNP12* | 2 | 7.7 | 2 | 3.4 | 18 | -0.7 | C |
| **Population** | **Chromosome** | **QTL** | **Trait** | **Location-Year** | **Marker** | **Pos., cM** | **LOD** | **Mean of Lillian allele** | **Mean of Vesper allele** | **PVE, %** | **Additive effect** | **Source of resistance allele** |
| V/L | 1B | *QLr.spa-1B* | IR | SC2014 | *wsnp_Ex_c1058_2020681* | 181.9 | 3 | 4.3 | 3.2 | 4.9 | 0.5 | V |
| V/L | 1B | *QLr.spa-1B* | S | SC2014 | *wsnp_Ex_c1058_2020681* | 181.9 | 2.2 | 9 | 4.6 | 3.7 | 2.2 | V |
| V/L | 1D | *QLr.spa-1D* | S | SC2013 | *Kukri_c2408_784* | 1 | 23.5 | 26.7 | 9.8 | 34.1 | 8.4 | V |
| V/L | 1D | *QLr.spa-1D* | IR | SC2013 | *Kukri_c2408_784* | 1 | 16 | 6.4 | 4 | 24.7 | 1.2 | V |
| V/L | 1D | *QLr.spa-1D* | S | SC2014 | *Kukri_c2408_784* | 1 | 13.2 | 11.9 | 1.7 | 20.1 | 5.1 | V |
| V/L | 1D | *QLr.spa-1D* | IR | SC2014 | *Kukri_c2408_784* | 1 | 15.8 | 4.9 | 2.6 | 23.5 | 1.2 | V |
| V/L | 1D | *QLr.spa-1D* | S | SC2015 | *Kukri_c2408_784* | 1 | 4.4 | 11.2 | 4.4 | 6.9 | 3.4 | V |
| V/L | 1D | *QLr.spa-1D* | IR | SC2015 | *Kukri_c2408_784* | 1 | 15.5 | 6.2 | 2.8 | 22.6 | 1.7 | V |
| V/L | 1D | *QLr.spa-1D* | S | MD2015 | *Kukri_c2408_784* | 1 | 22.7 | 47 | 12 | 31.1 | 17.5 | V |
| V/L | 1D | *QLr.spa-1D* | IR | MD2015 | *Kukri_c2408_784* | 1 | 19. 6 | 6 | 2.6 | 27.5 | 1.7 | V |
| V/L | 1D | *QLr.spa-1D* | S | LN2014 | *Kukri_c2408_784* | 1 | 21.9 | 2.8 | 0.2 | 30.4 | 1.3 | V |
| V/L | 4A | *QLr.spa-4A* | S | SC2013 | *wsnp_BF484585A_Td_2_1* | 73.3 | 3.8 | 14.3 | 20.5 | 4.1 | -3.1 | L |
| V/L | 4A | *QLr.spa-4A* | IR | SC2013 | *Ex_c70424_465* | 79.7 | 4.3 | 3.6 | 6.9 | 6.6 | -1.7 | L |
| V/L | 4A | *QLr.spa-4A* | S | SC2014 | *wsnp_BF484585A_Td_2_1* | 73.3 | 3 | 3.9 | 9.1 | 5 | -2.6 | L |
| V/L | 4A | *QLr.spa-4A* | IR | SC2014 | *wsnp_BF484585A_Td_2_1* | 73.3 | 4.4 | 3 | 4.3 | 6.6 | -0.7 | L |
| V/L | 4A | *QLr.spa-4A* | S | SC2015 | *Kukri_rep_c102142_525* | 77.8 | 4.7 | 2.8 | 12.3 | 7 | -4.7 | L |
| V/L | 4A | *QLr.spa-4A* | IR | SC2015 | *CAP11_c279_66* | 79 | 3.6 | 3.5 | 5.3 | 5.8 | -0.9 | L |
| V/L | 4A | *QLr.spa-4A* | S | MD2015 | *CAP11_c279_66* | 79 | 3.3 | 9.8 | 49.9 | 5.2 | -20 | L |
| V/L | 4A | *QLr.spa-4A* | IR | MD2015 | *CAP11_c279_66* | 79 | 3.6 | 2.2 | 6.4 | 5.6 | -2.1 | L |
| V/L | 4A | *QLr.spa-4A* | S | LN2014 | *wsnp_BF484585A_Td_2_1* | 73.3 | 6.14 | 0.7 | 2.2 | 9 | -0.8 | L |
| V/L | 6B | *QLr.spa-6B* | S | SC2013 | *BobWhite_c36415_378* | 118.6 | 3.8 | 22.4 | 15 | 6.6 | 3.7 | V |
| V/L | 6B | *QLr.spa-6B* | S | SC2014 | *BobWhite_c36415_378* | 118.6 | 4.7 | 10.2 | 3.9 | 7.7 | 3.2 | V |
| V/L | 6B | *QLr.spa-6B* | S | LN2014 | *BobWhite_c36415_378* | 118.6 | 3.9 | 2.1 | 1 | 6.2 | 0.6 | V |
| V/L | 7B | *QLr.spa-7B.1* | S | SC2013 | *Kukri_c109962_396* | 0 | 4.2 | 22.6 | 15.2 | 3.7 | 6.7 | V |
| V/L | 7B | *QLr.spa-7B.1* | IR | SC2013 | *Kukri_c109962_396* | 0 | 3 | 5.8 | 4.6 | 5.1 | 0.6 | V |
| V/L | 7B | *QLr.spa-7B.2* | S | SC2013 | *RFL_Contig71_386* | 232 | 4.9 | 23 | 14.8 | 7.7 | 4.1 | V |
| V/L | 7B | *QLr.spa-7B.2* | S | SC2014 | *RFL_Contig71_386* | 232 | 5.1 | 10.1 | 3.6 | 3.3 | 8.1 | V |
| V/L | 7B | *QLr.spa-7B.2* | S | SC2015 | *RFL_Contig71_386* | 232 | 4.8 | 11.4 | 4.4 | 7.3 | 3.5 | V |
| V/L | 7B | *QLr.spa-7B.2* | S | MD2015 | *RFL_Contig71_386* | 232 | 6.5 | 40.1 | 20.4 | 9.8 | 9.8 | V |
| V/L | 7B | *QLr.spa-7B.2* | IR | MD2015 | *RFL_Contig71_386* | 232 | 5 | 5.2 | 3.5 | 7.6 | 0.9 | V |
| V/L | 7D | *QLr.spa-7D* | S | SC2013 | *SNP12* | 7.5 | 5.9 | 13.5 | 22.7 | 9.9 | -4.6 | L |
| V/L | 7D | *QLr.spa-7D* | IR | SC2013 | *SNP12* | 7.5 | 10 | 4.1 | 6.1 | 16.3 | -1 | L |
| V/L | 7D | *QLr.spa-7D* | S | SC2014 | *BobWhite_c40479_283* | 0 | 3.4 | 4.1 | 9.6 | 5.7 | -2.7 | L |
| V/L | 7D | *QLr.spa-7D* | IR | SC2015 | *SNP12* | 7.5 | 3.1 | 3.6 | 5.2 | 5 | -0.8 | L |
| V/L | 7D | *QLr.spa-7D* | S | MD2015 | *SNP12* | 7.5 | 8.4 | 17.7 | 40.6 | 12.9 | -11.4 | L |
| V/L | 7D | *QLr.spa-7D* | IR | MD2015 | *SNP12* | 7.5 | 6.4 | 3.2 | 5.3 | 10 | -1 | L |
| **Population** | **Chromosome** | **QTL** | **Trait** | **Location-Year** | **Marker** | **Pos., cM** | **LOD** | **Mean of Stettler allele** | **Mean of Vesper allele** | **PVE, %** | **Additive effect** | **Source of resistance allele** |
| V/S | 1D | *QLr.spa-1D* | S | SC2014 | *BobWhite_c4303_524* | 58.6 | 5.1 | 7.3 | 1.2 | 22.4 | 3.1 | V |
| V/S | 1D | *QLr.spa-1D* | IR | SC2014 | *BobWhite_c4303_524* | 58.6 | 4.2 | 5.1 | 3.4 | 19.2 | 0.9 | V |
| V/S | 1D | *QLr.spa-1D* | S | LN2014 | *BobWhite_c4303_524* | 58.6 | 3.3 | 10.9 | 0.5 | 15.1 | 5.4 | V |
| V/S | 1D | *QLr.spa-1D* | S | SC2015 | *BobWhite_c4303_524* | 58.6 | 7.2 | 29.7 | 12.7 | 29.6 | 8.5 | V |
| V/S | 1D | *QLr.spa-1D* | IR | SC2015 | *BobWhite_c4303_524* | 58.6 | 4.6 | 4.6 | 2.4 | 23.5 | 1.1 | V |
| V/S | 1D | *QLr.spa-1D* | S | MD2015 | *BobWhite_c4303_524* | 58.6 | 12.5 | 54.7 | 17 | 44.8 | 18.9 | V |
| V/S | 1D | *QLr.spa-1D* | IR | MD2015 | *BobWhite_c4303_524* | 58.6 | 6.7 | 7 | 4 | 28 | 1.5 | V |
| V/S | 2A | *QLr.spa-2A.2* | S | MD2015 | BS00022393_51 | 50.1 | 3.5 | 46.5 | 24.1 | 15.7 | 11.2 | V |
| V/S | 2A | *QLr.spa-2A.2* | IR | MD2015 | BS00022393_51 | 50.1 | 3.2 | 6.5 | 4.2 | 14.4 | 1.1 | V |
| V/S | 2A | *QLr.spa-2A.2* | IR | SC2014 | BobWhite_c2022_245 | 82.3 | 3.4 | 5 | 3.6 | 13.6 | 0.7 | V |
| **Population** | **Chromosome** | **QTL** | **Trait** | **Location-Year** | **Marker** | **Pos., cM** | **LOD** | **Mean of Red Fife allele** | **Mean of Stettler allele** | **PVE, %** | **Additive effect** | **Source of resistance allele** |
| S/ R | 2D | *QLr.spa-2D.2* | S | SC2014 | *Kukri_rep_c105822_804* | 59.076 | 3.3 | 9 | 6.8 | 10.5 | 1.1 | S |
| S/R | 2D | *QLr.spa-2D.2* | S | LN2014 | *Kukri_rep_c105822_804* | 59.076 | 6.8 | 1 | 0.1 | 13.3 | 0.5 | S |
| S/R | 2D | *QLr.spa-2D.2* | S | SC2015 | *Kukri_rep_c105822_804* | 59.076 | 12.9 | 13.1 | 3.4 | 24 | 4.8 | S |
| S/R | 2D | *QLr.spa-2D.2* | IR | SC2015 | *Kukri_rep_c105822_804* | 59.076 | 14.3 | 4.9 | 2.9 | 26.2 | 1 | S |
| S/R | 2D | *QLr.spa-2D.2* | S | MD2015 | *Kukri_rep_c105822_804* | 59.076 | 21.1 | 52.9 | 20.9 | 36.1 | 16 | S |
| S/R | 2D | *QLr.spa-2D.2* | IR | MD2015 | *Kukri_rep_c105822_804* | 59.076 | 22.5 | 6.7 | 3.4 | 37.9 | 1.7 | S |
| S/ R | 6B | *QLr.spa-6B* | S | SC2015 | *BS00010993_51* | 111 | 3.9 | 11.6 | 6.1 | 8 | 2.8 | S |
| S/ R | 7A | *QLr.spa-7A* | S | MD2015 | *tplb0031i24_1212* | 4.412 | 5.6 | 30.8 | 48.6 | 11.2 | -8.9 | R |
| S/ R | 7A | *QLr.spa-7A* | IR | MD2015 | *tplb0031i24_1212* | 4.412 | 4.1 | 4.6 | 6.1 | 8.4 | -0.8 | R |
| S/ R | 7B | *QLr.spa-7B.2* | S | LN2014 | *BS00108630_51* | 166 | 3.1 | 0.3 | 0.9 | 6.2 | -0.3 | R |

^a^ Population names: C/Cd, Carberry/AC Cadillac; C/V, Carberry/Vesper; V/L, Vesper/Lillian; V/S, Vesper/Stettler; S/R, Stettler/Red Fife.

^b^Leaf rust trait, S, severity; IR, infection response.

^c^ Location names followed by the last two digits of test years: SC, Swift Current; BD, Brandon; MD, Morden; LN, Lincoln.

^d^LOD, logarithm of odds score.

^e^PVE, percent phenotypic variance explained by individual QTL.
