# Supplementary material for: Mapping quantitative trait loci associated with leaf rust resistance in five spring wheat populations using single nucleotide polymorphism markers
Source: PLoS One. 2020 Apr 8;15(4):e0230855. doi: 10.1371/journal.pone.0230855 (PMC7141615; doi:10.1371/journal.pone.0230855)
Supplement: S8 Table — (DOCX) [file pone.0230855.s009.docx]

**S8 Table. Location of the leaf rust resistance QTL associated markers in the** [**International Wheat Genome Sequencing Consortium (IWGSC)**](https://www.wheatgenome.org/) **RefSeq. V. 1.0 wheat genome assembly.**

| **QTL** | **Closest marker** | **Start, bp** | **End, bp** | **Length, bp** |
| --- | --- | --- | --- | --- |
| ***Carberry/AC Cadillac*** |  |  |  |  |
| *QLr.spa-1A* | *IACX1465* | 33,375,718 | 33,375,807 | 89 |
| *QLr.spa-2A.1* | *BS00041816_51* | 611,316,317 | 611,316,418 | 101 |
| *QLr.spa-2B.1* | *Excalibur_c39493_251* | 6,210,270 | 6,210,371 | 101 |
| *QLr.spa-2B.2* | *Kukri_c53810_137* | 31,622,692 | 31,622,791 | 99 |
| *QLr.spa-2D.1* | *Ex_c2115_3369* | 435,045,121 | 435,045,222 | 101 |
| *QLr.spa-3B* | *Tdurum_contig79629_538* | 10,956,617 | 10,956,718 | 101 |
| *QLr.spa-4B.1* | *Tdurum_contig12204_1131* | 705,442,971 | 705,443,072 | 101 |
| *QLr.spa-4B.2* | *BS00021984_51* | 35,520,721 | 35,520,822 | 101 |
| *QLr.spa-5A* | *BobWhite_c1387_798* | 528,114,516 | 528,114,617 | 101 |
| *QLr.spa-6A* | *BobWhite_c39821_195* | 772,899,487 | 772,899,578 | 91 |
| *QLr.spa-7A* | *BS00063860_51* | 132,308,499 | 132,308,600 | 101 |
| *QLr.spa-7B.1* | *Ex_c101666_634* | 24,060,478 | 24,060,563 | 85 |
| *QLr.spa-7B.2* | *RAC875_c57326_85* | 702,975,870 | 702,975,971 | 101 |
| ***Carberry/Vesper*** |  |  |  |  |
| *QLr.spa-1D* | *RAC875_c2070_566* | 417,148 | 417,249 | 101 |
| *QLr.spa-2A.2* | *Kukri_c46040_620* | 12,631,079 | 12,631,164 | 85 |
| *QLr.spa-2B.1* | *BobWhite_c12144_216* | 10,786,105 | 10,786,172 | 67 |
| *QLr.spa-7A* | *BS00053365_51* | 692,907,342 | 692,907,436 | 94 |
| ***Vesper/Lillian*** |  |  |  |  |
| *QLr.spa-1B* | *wsnp_Ex_c1058_2020681* | 678,317,710 | 678,317,911 | 201 |
| *QLr.spa-1D* | *Kukri_c2408_784* | 1,342,642 | 1,342,743 | 101 |
| *QLr.spa-4A* | *Ex_c70424_465* | 476,980,504 | 476,980,584 | 80 |
| *QLr.spa-6B* | *BobWhite_c36415_378* | 405,664,842 | 405,664,943 | 101 |
| *QLr.spa-7B.1* | *Kukri_c109962_396* | 24,061,366 | 24,061,453 | 87 |
| *QLr.spa-7B.2* | *RFL_Contig71_386* | 741,718,414 | 741,718,515 | 101 |
| ***Vesper/Stettler*** |  |  |  |  |
| *QLr.spa-1D* | *BobWhite_c4303_524* | 3,448,803 | 3,448,903 | 100 |
| *QLr.spa-2A.2* | *BS00022393_51* | 5,929,806 | 5,929,907 | 101 |
| ***Stettler/Red Fife*** |  |  |  |  |
| *QLr.spa-2D.2* | *Kukri_rep_c105822_804* | 97,949,792 | 97,949,893 | 101 |
| *QLr.spa-6B* | *BS00010993_51* | 662,257,779 | 662,257,880 | 101 |
| *QLr.spa-7A* | *tplb0031i24_1212* | 723,296,502 | 723,296,596 | 94 |
| *QLr.spa-7B.2* | *BS00108630_51* | 714,050,497 | 714,050,598 | 101 |
