# Supplementary material for: Mapping quantitative trait loci associated with leaf rust resistance in five spring wheat populations using single nucleotide polymorphism markers
Source: PLoS One. 2020 Apr 8;15(4):e0230855. doi: 10.1371/journal.pone.0230855 (PMC7141615; doi:10.1371/journal.pone.0230855)
Supplement: S1 Fig — Some markers were removed from the linkage maps illustrated here for simplification of the presentation. The test environments are denoted by the test years preceded by an abbreviation of the location as follows: MD, Morden; BD, Brandon; SC, Swift Current, Canada, and LN, Lincoln, New Zealand. The disease phenotypes are abbreviated: LRSEV, leaf rust severity, and LRIR, leaf rust infection response. Chromosome names are followed by population name abbreviations: CCd, Carberry/AC Cadillac; CV, Carberry/Vesper; VL, Vesper/Lillian; VS, Vesper/Stettler; RS, Red Fife/Stettler. (DOCX) [file pone.0230855.s010.docx]

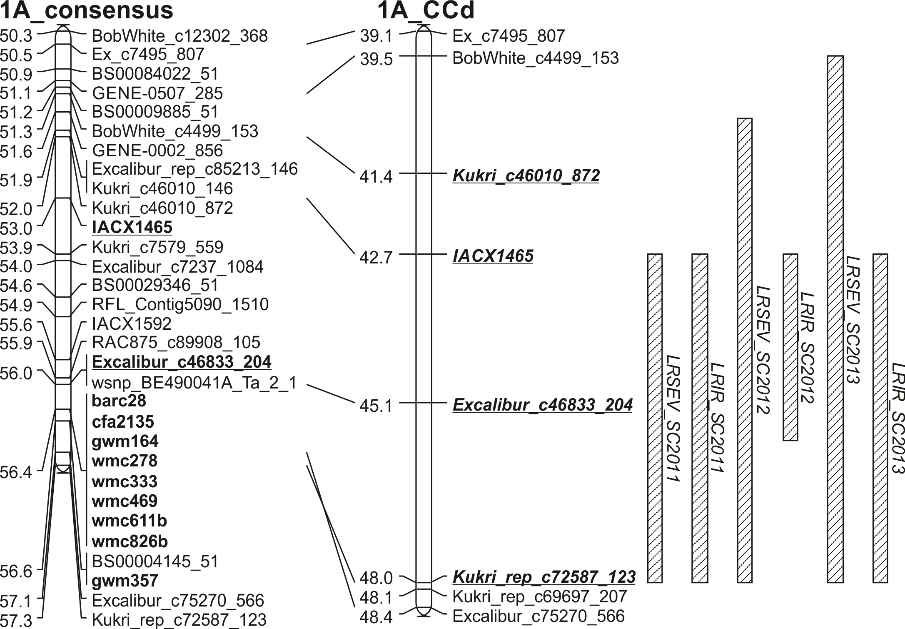

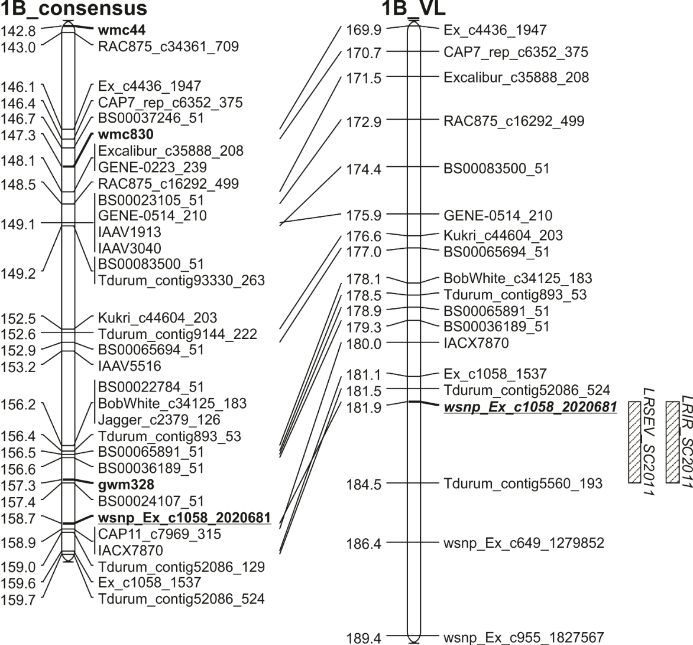


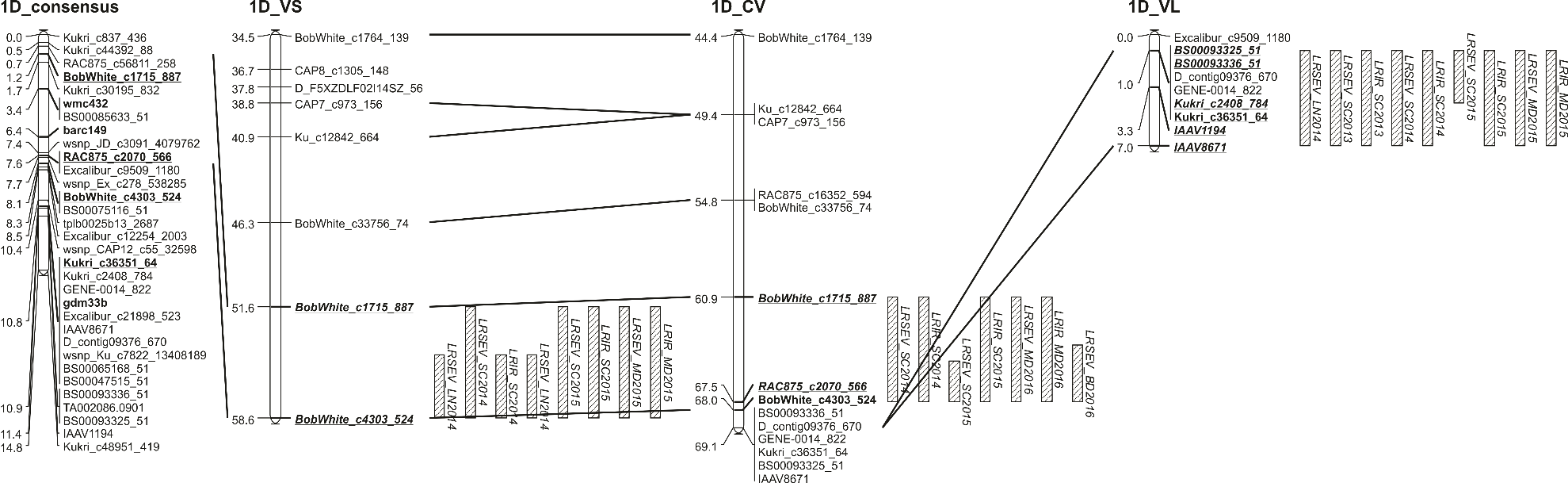


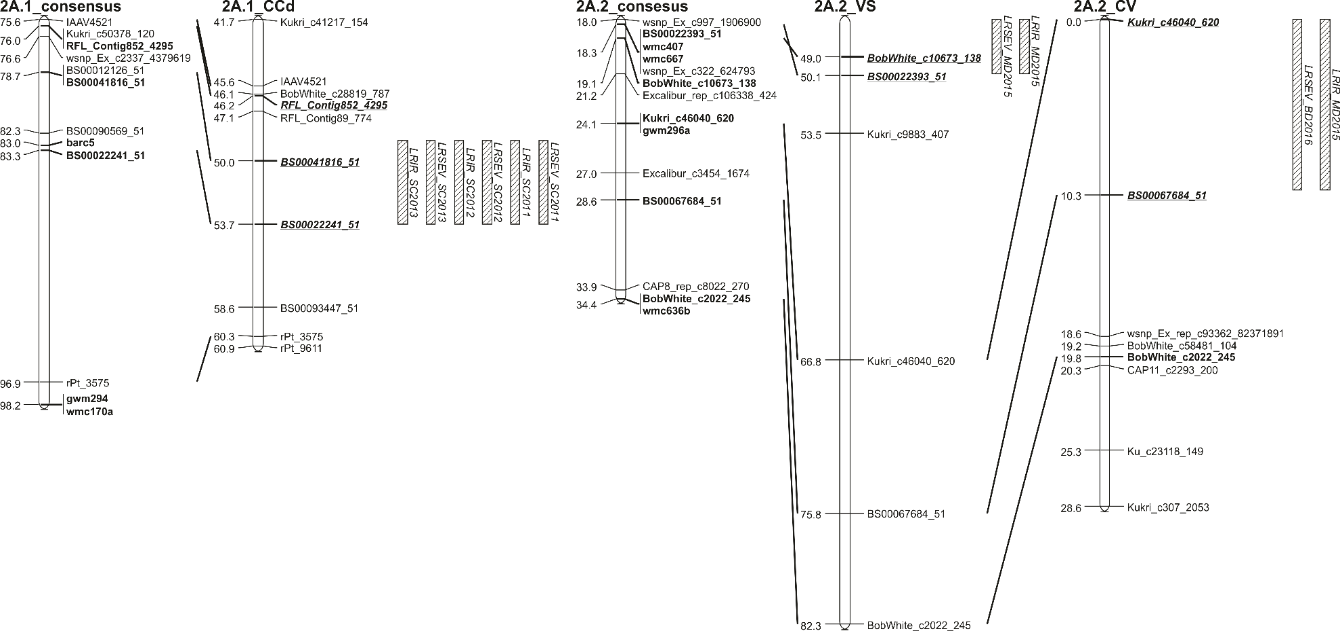


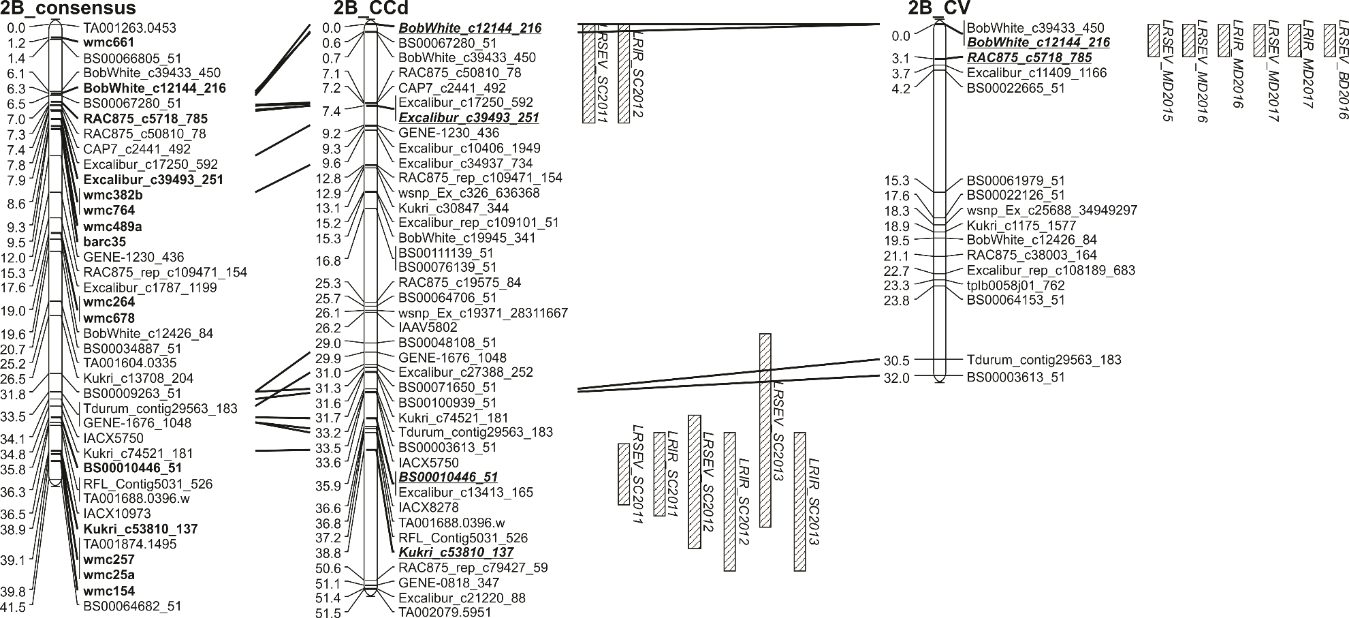


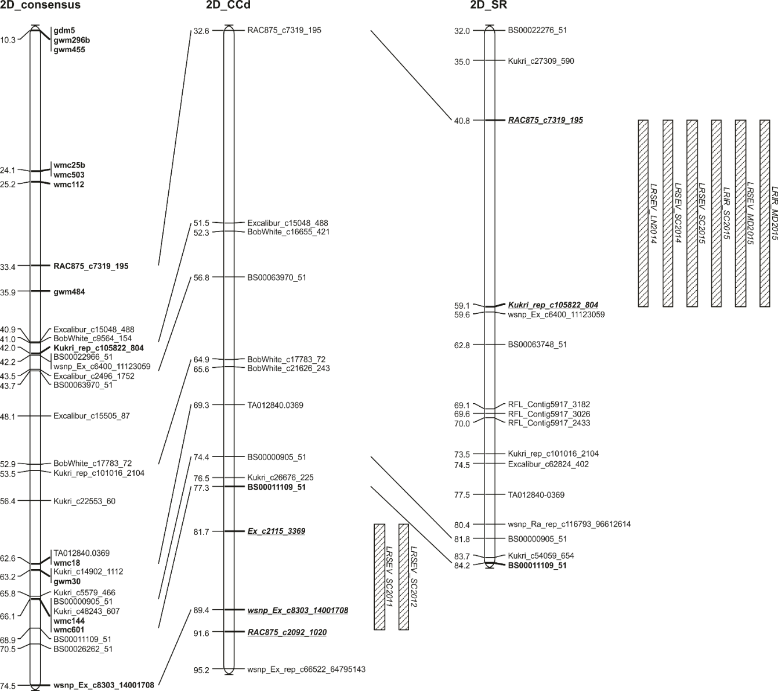

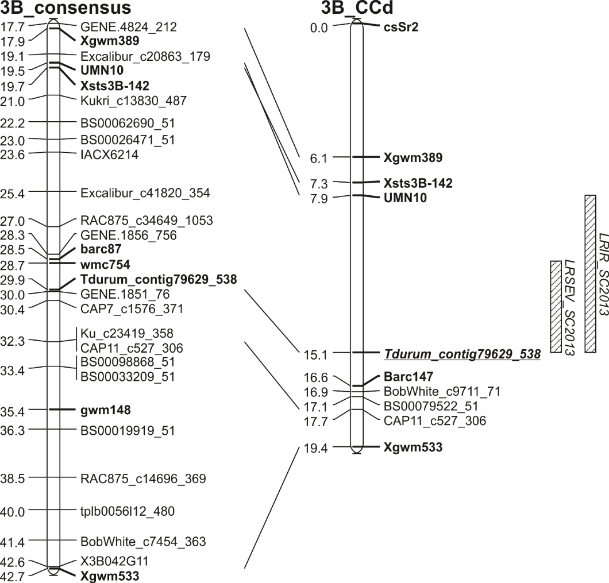

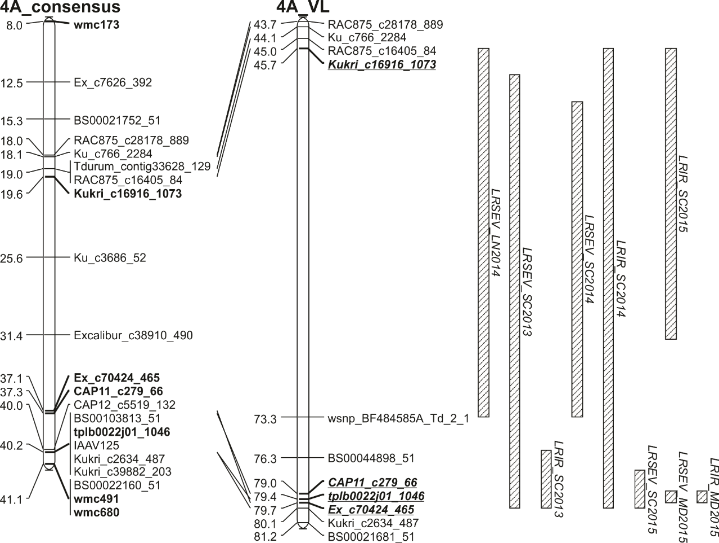

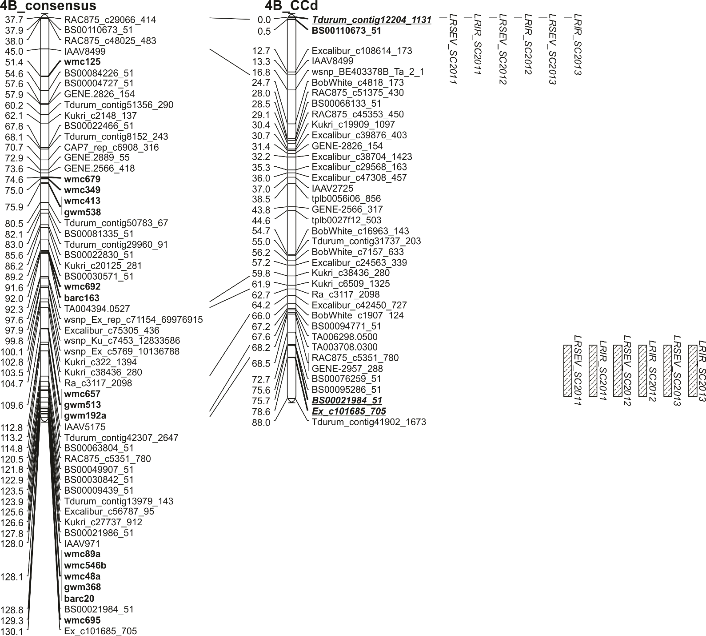


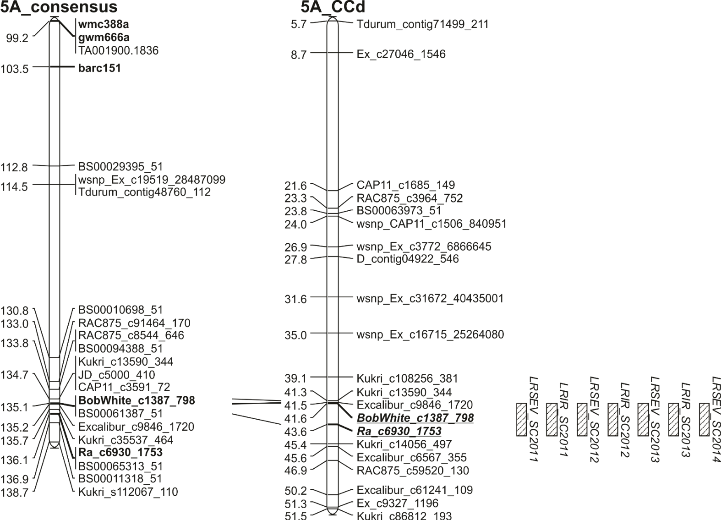

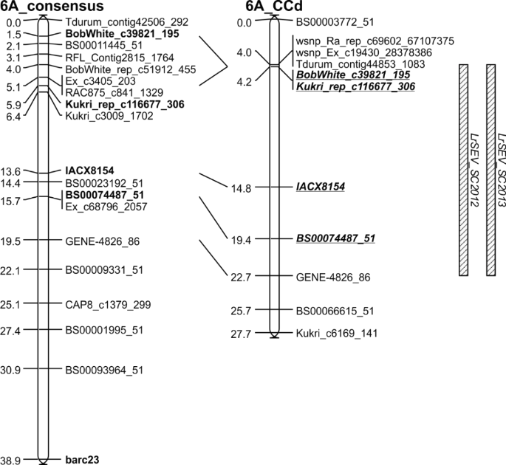


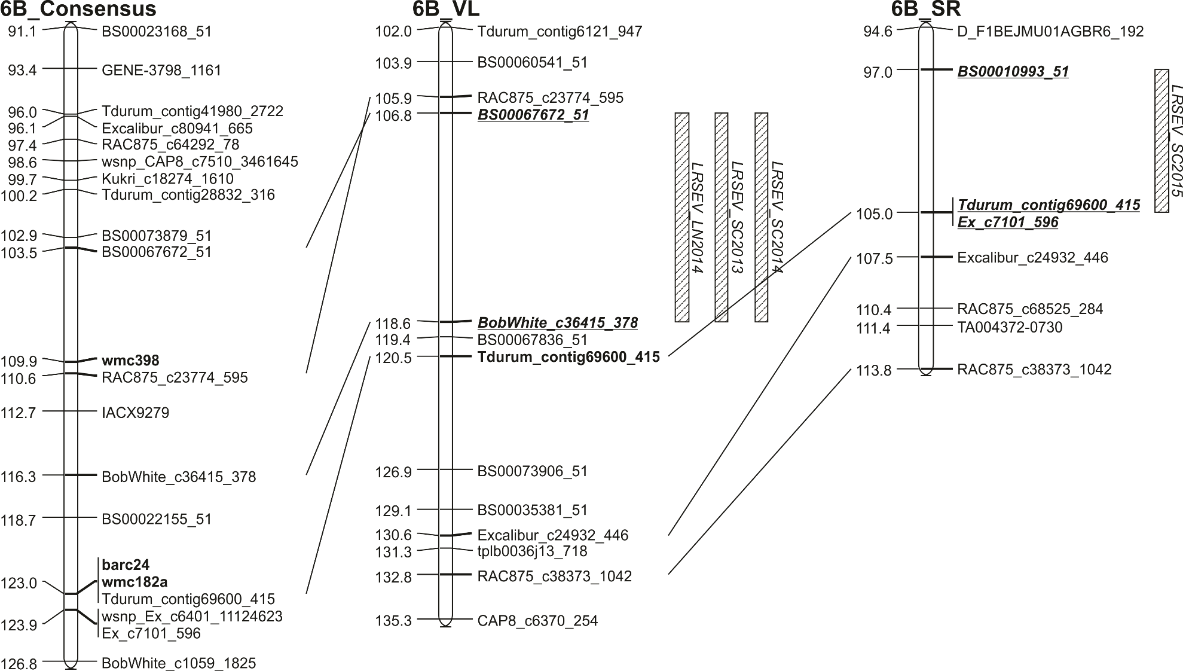


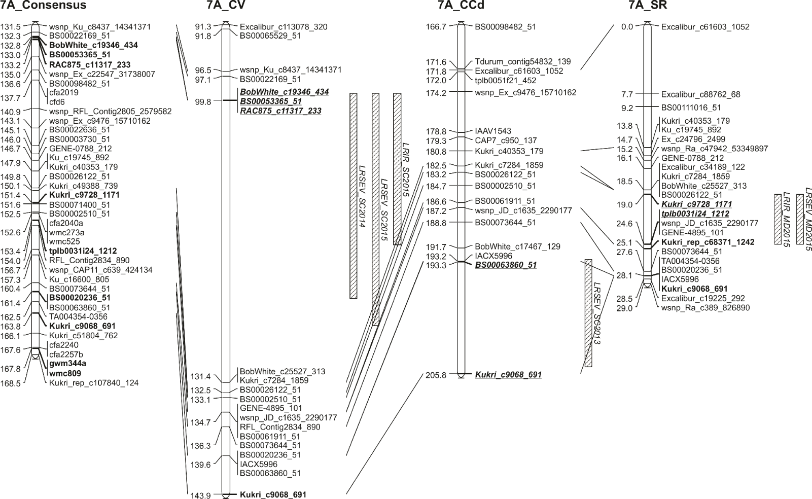


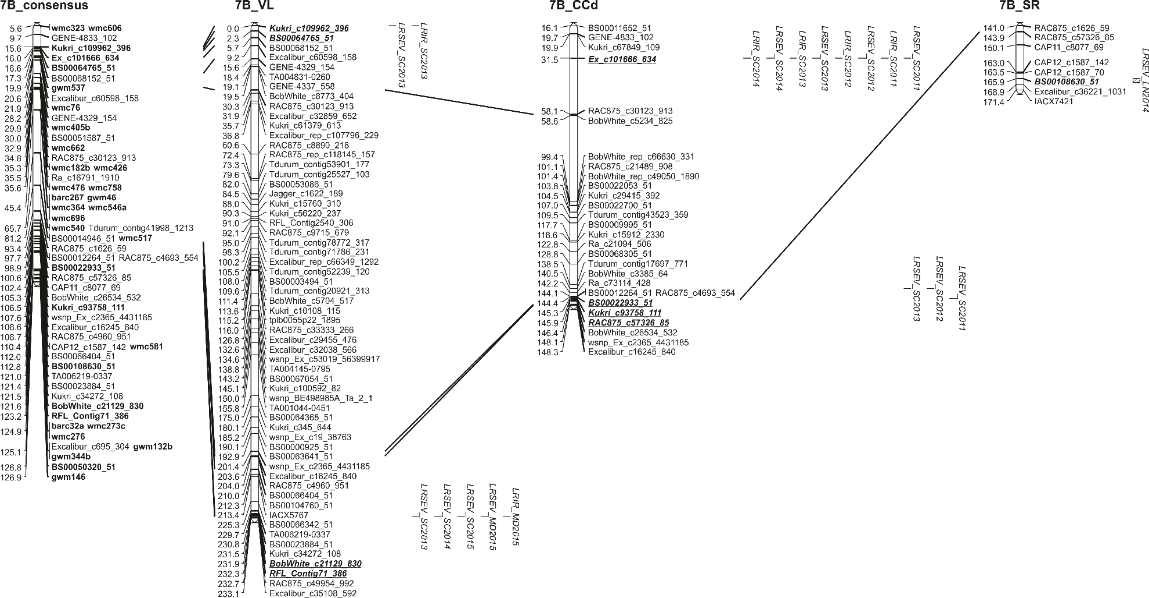


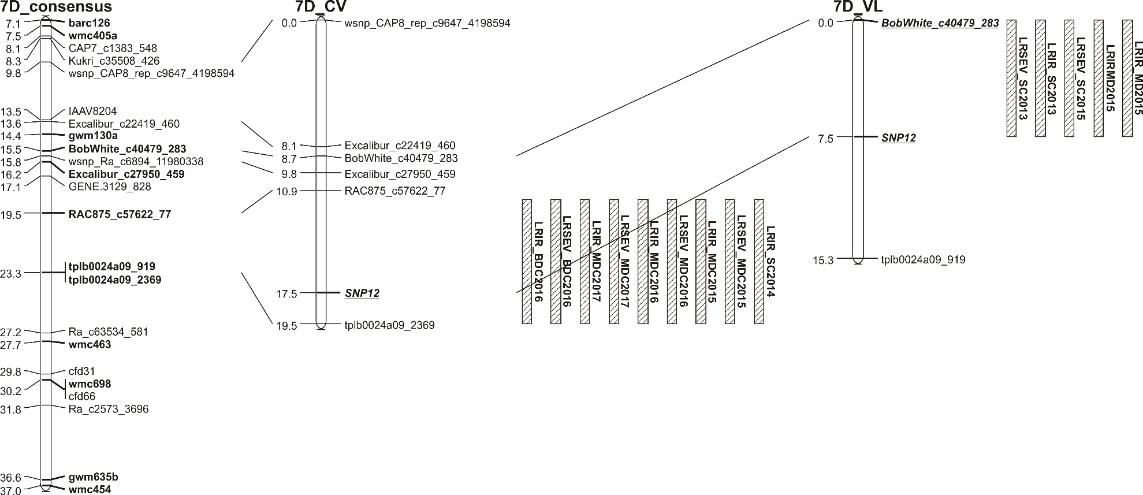


**S1 Fig. Partial genetic linkage map of quantitative trait loci for resistance to leaf rust in five different wheat populations aligned with the hexaploid wheat consensus map constructed using SSR and SNP markers. Some markers were removed from the linkage maps illustrated here for simplification of the presentation. The test environments are denoted by the test years preceded by an abbreviation of the location as follows: MD, Morden; BD, Brandon; SC, Swift Current, Canada, and LN, Lincoln, New Zealand. The disease phenotypes are abbreviated: LRSEV, leaf rust severity, and LRIR, leaf rust infection response. Chromosome names are followed by population name abbreviations: CCd, Carberry/AC Cadillac; CV, Carberry/Vesper; VL, Vesper/Lillian; VS, Vesper/Stettler; RS, Red Fife/Stettler.**
